# Face the Edges: Catalytic Active Sites of Nanomaterials

**DOI:** 10.1002/advs.201500085

**Published:** 2015-06-10

**Authors:** Bing Ni, Xun Wang

**Affiliations:** ^1^ Department of ChemistryTsinghua UniversityBeijing100084P. R. China

**Keywords:** nanomaterial morphology, catalytic active centers, interfaces, nanoframes, ultrathin nanowires

## Abstract

Edges are special sites in nanomaterials. The atoms residing on the edges have different environments compared to those in other parts of a nanomaterial and, therefore, they may have different properties. Here, recent progress in nanomaterial fields is summarized from the viewpoint of the edges. Typically, edge sites in MoS_2_ or metals, other than surface atoms, can perform as active centers for catalytic reactions, so the method to enhance performance lies in the optimization of the edge structures. The edges of multicomponent interfaces present even more possibilities to enhance the activities of nanomaterials. Nanoframes and ultrathin nanowires have similarities to conventional edges of nanoparticles, the application of which as catalysts can help to reduce the use of costly materials. Looking beyond this, the edge structures of graphene are also essential for their properties. In short, the edge structure can influence many properties of materials.

## 1 Introduction

This is an open access article under the terms of the Creative Commons Attribution License, which permits use, distribution and reproduction in any medium, provided the original work is properly cited.

Tailoring the morphologies of nanomaterials has achieved great success in catalyst optimization. What are the key factors to tune the catalytic properties? The answers may be complicated. From the view of band theory, the morphology varies the energy bands of particles.^1^ From the point of chemistry, size effect in performance is related with higher content of surface atoms.^2^ However, these kinds of explanations are not always suitable in every case. Band theory is usually applied in reactions with light as the energy input.^3^ Particles with smaller size possess larger amount of surface atoms, however, it is not always true that smaller particles show better performance.^4^ Unveiling the mystery underneath the high catalytic activity is a tough but meaningful and valuable work. From the view of geometry, any particles are enclosed by several facets, the intersection of facets is an edge, and the intersection of two edges is a corner. Tuning the morphology is actually tuning the facets, edges and corners. There are many reviews concerning the facets of nanoparticles as key points for properties,^5^, ^6^, ^7^ however, the edges are less discussed.

Atoms on edges usually show different properties comparing with atoms on inner faces (here “inner” refers to “on the surface but not on the edge”). Increasing the content of edge sites may help to further optimize the activities for catalysts. It is well accepted that edges of MoS_2_, other than basal plane, are active for many catalytic reactions.^8^, ^9^ This is not an isolate case, and the edges of noble metals are also linked with high activities. Looking beyond this, the edge structure of graphene plays a vital role for determining the energy band and other related properties.^10^ Here we consider that the edges are keys for catalytic reactions to organize the review. At first, we begin with an overall picture of nanoparticles (NPs) that serve as catalysts, then several examples of conventional edges in nanomaterials (which refer to natural boundaries of surfaces) as active sites are presented in section 3. Here we mainly discuss reactions such as hydrogen evolution reaction, which is important for energy conversion, and CO_2_ reduction, which is essential for environment protection. We do not limit other scope in homogeneous NPs, the edge sites in heterostructures are more intricate and an introduction to these is presented. Many catalytic reactions require cooperation between various components, thus the activities may be ascribed to the novel interface. However, the lattices on both sides of the interface would greatly hinder the approach of substrates, thus almost no activity could be found at the interior interface. This is to say that the edges of interface are pivotal for activities, so we also review this area. There are also other kinds of edge structures which cannot be simply explained as natural boundary of surfaces. If the edges of NPs are active for applications, an intuitional method to reduce the use of expensive catalyst is to construct nanoframes (NFs) which only contain edges. NFs, together with ultrathin nanowires (UNWs), are considered as atypical edges of novel structures in this review. Untangling the intersecting lines (edges) in NFs can lead to nanowires (NWs). UNWs are an emerging hot topic of material fabrication and applications. However, the detailed structures of UNWs are far from well‐known, so it is hard to determine whether UNWs could resemble conventional edges in nanoparticles or not now. But with the fast developing speed of high resolution and non‐destructive analytic methods, we should be confident that the answer would be soon solved. In the sections afterward, we present some areas concerning edges not only for the application as catalyst, edges of graphene could determine various properties. The researches on their atomic structures may shed light on research of other materials' edge structures. In conclusion, there are many types of edge structures, a comprehensive review of them could help with their applications.

## 2 Catalysts and Nanoparticles

Chemical reactions happen almost everywhere from life to industry. Among them, catalysts play vital roles in most situations. Active enzymes which could accelerate reactions in cells ensure that organs could function. Industrial catalysts from petroleum refining to car exhaust catalysts are key steps to convenient modern life. Catalysts are also essential in academic research. Proper catalysts could help to activate C‐H bonds, couple new C‐C bonds in desired position, and alter function groups.^11^ These kinds of reactions are usually achieved via the introduction of soluble metal complex in the same phase with the reactants, which are called homogeneous catalysts. Homogeneous catalysts have a number of advantages such as high selectivity, good yield and easy to optimize. The active center of homogeneous catalysts is always related with the metal center, and its coordinated environments, which make them easy to optimize. However, catalysts for industry also require easy recovery or recycle. Many homogeneous catalytic systems cannot be commercialized because of the difficulties encountered in separating the catalyst from the final products.^12^ Otherwise the metal contamination could affect the safety of products, especially for the pharmaceutical industry. This disobeys the requirements of green chemistry.^13^

Heterogeneous catalysts that react with reactants in different phases might be a promising method to overcome the obstacle of separation while maintaining the yield of products. In fact, there are many important industrial homogeneous catalysts in biphasic systems,^14^ which are fixed on supports (usually are “metal on support” catalysts), in order to suit the demand of easy separation. Another hot topic of heterogeneous catalysts is using NPs to serve as highly active catalysts.^15^ Academic researches of heterogeneous catalysis aim to realize undeveloped reactions^16^, ^17^ or optimize the activities of developing reactions.^18^, ^19^, ^20^ The boundary between homogeneous or heterogeneous is the size of catalyst. While the functional dimension of a chemical bond in reaction typically has a length of about 0.2 nm, a larger catalyst would not do much help to combine atoms which are too far from each other if the migration of atoms are hampered, which is usually true in real reactions.^21^

The catalysis effect rellies on the lowering of activation energy, by altering the intermediated steps of reaction. This process usually involves the binding of substrates or intermediates with catalysts, the release of the following intermediates or products and the recycle of catalysts. When the proper metal core is selected, optimization of homogeneous catalyst relies mainly on altering the ligands around metal to tune the electronic structure to achieve suitable binding or releasing. For “metal on support” catalyst, to tune the performance requires the optimization of metals and selection of supports, while the supports are not innocent during catalytic reactions in many cases.^22^, ^23^, ^24^ For NPs as catalysts, the optimizations of their performances are mainly achieved by tailoring their morphologies. Energy band of crystal stems from the Bragg diffraction of electron, thus tuning the sizes and morphologies could tune the electronic structures of nanocrystals and influence the activities. On the other hand, the large amount of surface atoms could enhance the regional catalyst concentration. As researches go deeper, cooperation between various metals are found to be important in reactions.^25^ Many natural enzymes possess multi‐metal core, such as nitrogenase.^26^ The cooperation of various metals is also true in organic synthesis.^27^ NPs feature vast atoms on surface, cooperation between them are inevitable, and the distance between surface atoms could be tailored by exposing different facets. So using NPs as catalyst are easy to optimize, thus they are promising for application.

However, there are still some obstacles hindering the realization of NPs as high efficient catalysts. First of all, the capping agents on NPs' surfaces, which are used in most NP synthesis, may prevent the contact between reactant and particles, thus lowering the efficiency. Secondly, the deterioration of NPs may hamper the recycle of catalysts. When noble metals are used as catalysts, the internal atoms seem useless in reaction, however, increase the total cost of materials. Last but not the least, the real mechanisms of reactions are unknown in many cases, thus add extra difficulties for optimizing. In short, the active center of NPs need to be debunked. With the knowledge of active center, rational optimization or design of high efficient catalysts is not impossible.

When NPs are used as catalysts, the surface atoms are usually considered as active centers. They are able to contact and combine with substrates, and reactions may happen at surface under suitable conditions. Thus the manner that substrates bind with NPs may influence the yield and selectivity of reactions.^28^ The surface structure of NPs could also affect the contact with substrate.^29^ The composition of composite NPs also complicates the detailing of active centers. These factors make the study of active centers more complicated.

To combine with substrates, the surface atoms must have unsaturated coordination, the coordination number (CN) may dramatically influence performance. Edges and corners of NPs feature lower CN than atoms on the surface, thus increasing the amount of them can help to tune the activity. Considering the very low content of corner sites of NPs, edges are better solutions to enhance the activities of catalysts. On the other hand, the chemical environment of edges in nanosheets or multicomponent interfaces is different with other parts, thus could be utilized to tune the electronic structure, as well as catalytic performance.

## 3 Edges as Active Sites

Here we present some prominent works that utilize the edges of nanomaterials as active centers, the optimization of edge structure could greatly enhance the performance of materials. In this section, we introduce three types of conventional edges in nanostructures, and a brief summary of them is presented in **Table** **1**. Taking MoS_2_ nanosheets as an example of 2D structure, the edges of them mainly refer to the boundary of sheets, and their chemical environments are different with atoms on basal plane because of the termination of atoms. The unique structure makes edges of 2D materials special and could be utilized as catalyst. For NPs, edges are the intersections of surfaces, they feature low CN and are active to bind some of the substrates or intermediates in catalytic reactions. As for the edges of multicomponent interface, they are specialized as the external boundary of conjunction of different components. The contact of two particles makes a two dimensional interface, so the edges of them may feature similar characteristics with 2D structures, such as a special chemical environment. The “basal plane” of interface is not approachable due to the existence of particles, thus cannot simply be used as active sites. The more details and methods to enhance the relative catalytic performance of these three types of edges are present in the following discussions.

**Table 1:** A brief comparison of conventional edges

	2D structure	Nanoparticles	Multicomponent interface
Structural feature	The boundary of sheet	The intersection of surfaces	The external boundary of conjunction of different components
Chemical feature	Special chemical environment	Low CN	Special chemical environment
Method to enhance catalytic properties	Tune the structure; expose more edges	Enlarge the edge content	Select proper components; tune the conjunction manner; optimize the edge content

### 3.1 MoS_2_ and Hydrogen Evolution Reaction

**Figure 1:**
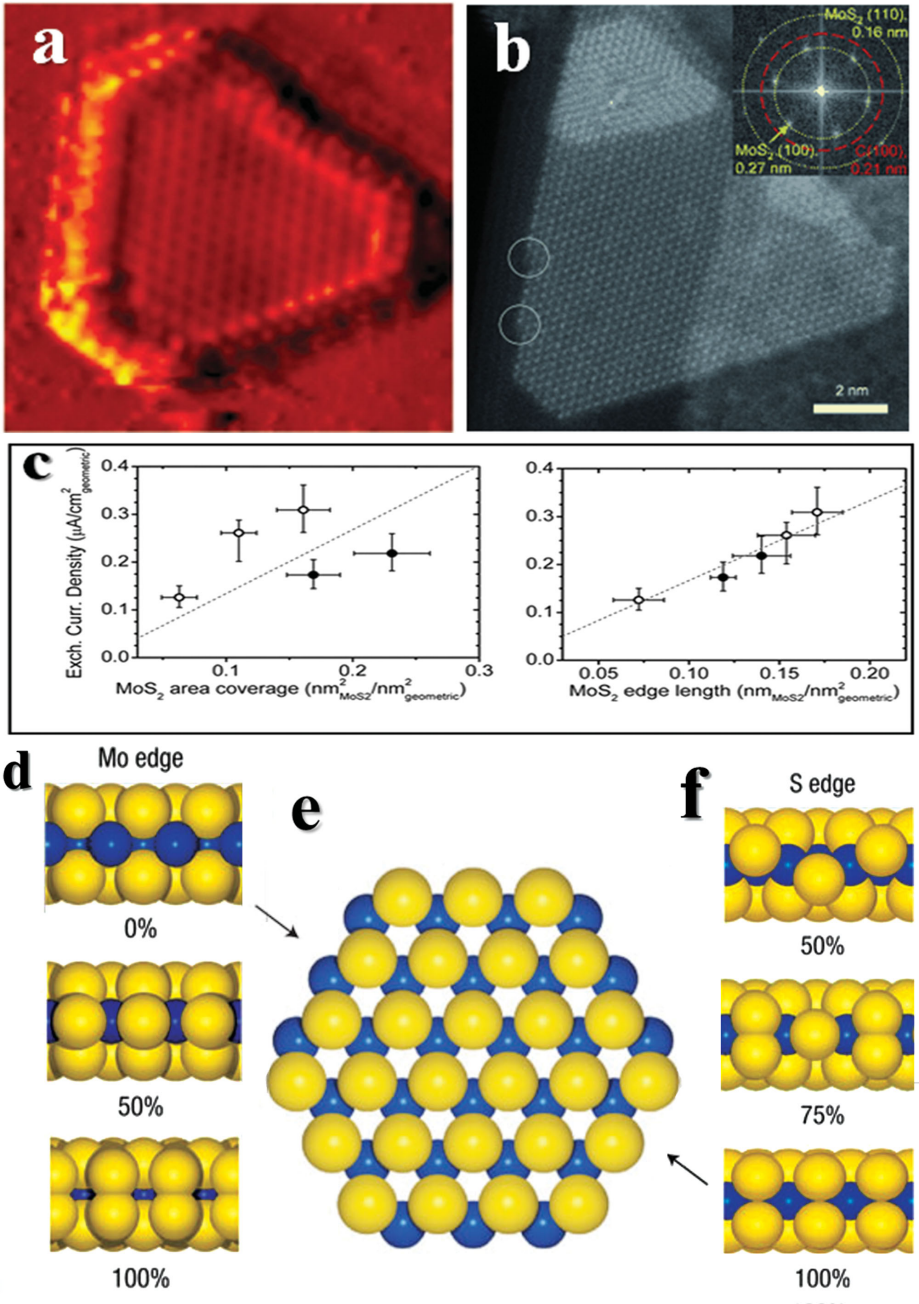
The structures of MoS_2_ and their activities for HER. a) Atomically resolved MoS_2_ particle by STM and c) their exchange current density versus MoS_2_ area coverage (left) or MoS_2_ edge length (right). In both figures, open circles and filled circles are samples prepared under different conditions. The exchange current density does not correlate with the area coverage of MoS_2_, whereas it shows a linear dependence on the MoS_2_ edge length. Reproduced with permission.^36^ Copyright 2007, American Association for the Advancement of Science. b) High‐resolution STEM image of an industrial MoS_2_ supported on a graphite support. The insert is a fast Fourier transform (FFT) of the image and shows hexagonally arranged spots at the 0.27 nm and 0.16 nm lattice distances, corresponding to the MoS_2_ (100) and (110) lattice planes, respectively. Moreover, a hexagonal set of lattice distances at 0.21 nm, corresponding to graphite (100), is also revealed. At the low‐indexed edges, kink sites may be present as indicated by white circles. The observation matches the 50% sulfur covered Mo edge structure. Reproduced with permission.^39^ d) Side view of the Mo edge, showing the fully stripped 0% edge and two almost equally stable edge configurations with 50% or 100% sulphur coverage, respectively. e) Ball model of the hexagonal MoS2 nanocluster. f) Side view of the S edge, showing three configurations representing 50%, 75% or 100% sulphur coverage, respectively. Reproduced with permission.^42^ Copyright 2007, Macmillan Publishers Ltd.

Searching for cheap and high efficient catalysts of hydrogen evolution reaction (HER) is required for water splitting and energy storage,^30^ lots of experimental^31^ and computational^32^ works have been done in this area. The noble metal, Pt, shows perfect activity for HER, however it is expensive and scarcity on earth. Meanwhile some natural enzymes which contains much less noble metals are also effective HER catalysts.^33^, ^34^ By comparing the active center of nitrogenase with different inorganic compounds, the Nørskov group found that the MoS_2_ edge possess similar structure and was chosen as a candidate for HER.^35^ From then on, vast researches of MoS_2_ for HER have been carried out to identify the active sites and further enhance the activities. In order to figure out the active sites, a combination of surface sensitive methods and reactivity studies must be sophisticatedly designed.^36^ Fresh and clean MoS_2_ polygonal single layer sheets with different sizes and area coverages could be obtained by a physical vapor deposition and followed by annealing.^37^ After characterizing by scanning tunneling micro­scopy (STM) under ultrahigh vacuum chamber (**Figure** **1**a) to get structure information, the products was tested for HER activity respectively. Figure 1c is the activities indicated by the exchange current density, larger value indicates a better performance. For sheets with different sizes, the exchange current densities are quite different, however it shows almost no relationship between basal sizes and activities, the reason is that the basal plane of MoS_2_ has no catalytic activity.^8^ By using edge length as the abscissa, a clear linear relationship appears, higher edge length shows higher activity. This direct and well‐designed experiment clearly identified that the edge of MoS_2_ is activity sites for HER.

It is well known that enzymes must adopt a specific configuration to achieve efficient catalytic performances, unravelling details of MoS_2_ edge structures would gain our knowledge to better understand the reactions. 2D MoS_2_ nanosheets can exist in various polymorphs, natural MoS_2_ favors an edge‐sharing MoS_6_ trigonal prisms,^38^ named 2H phase. For such configuration, two types of low‐index edge terminations are possible, one is (10$\bar{1}$0) Mo and the other is ($\bar{1}$010) S edges (Figure 1d‐f), but a triangular product implies only one edge type is energetically favored in fact. As for industrial‐style MoS_2_, high resolution scanning transmission electron microscopy (STEM) reveals the nanocatalyst suits the 50% sulfur cover Mo edge structures (Figure 1b).^39^ The edges of single‐layer MoS_2_ have two localized metallic states which indicate subtle electronic structure changes close to the edges,^40^ and result in the light lines in STM images. More STM studies reveal the coordination structures of Mo‐S at edge are a little different with bulk MoS_2_, a so‐called “S2 dimer” is formed by two outermost S atoms which contract in the direction perpendicular to the basal plane.^41^ Not to our surprise, the structures of MoS_2 ­_ with different sizes are not exact the same, they possess different Mo or S edge coverage. A rearrangement of the S atoms at edges would influence bonding strength of Mo‐S, thus changes the stability and catalytic activity.^42^ Another phase of MoS_2_ is called 1T phase, which is constructed by edge‐sharing MoS_6_ octahedra. It is a metastable structure, and can be prepared by ultrasound‐promoted hydration of lithium‐intercalated compounds.^43^

**Figure 2:**
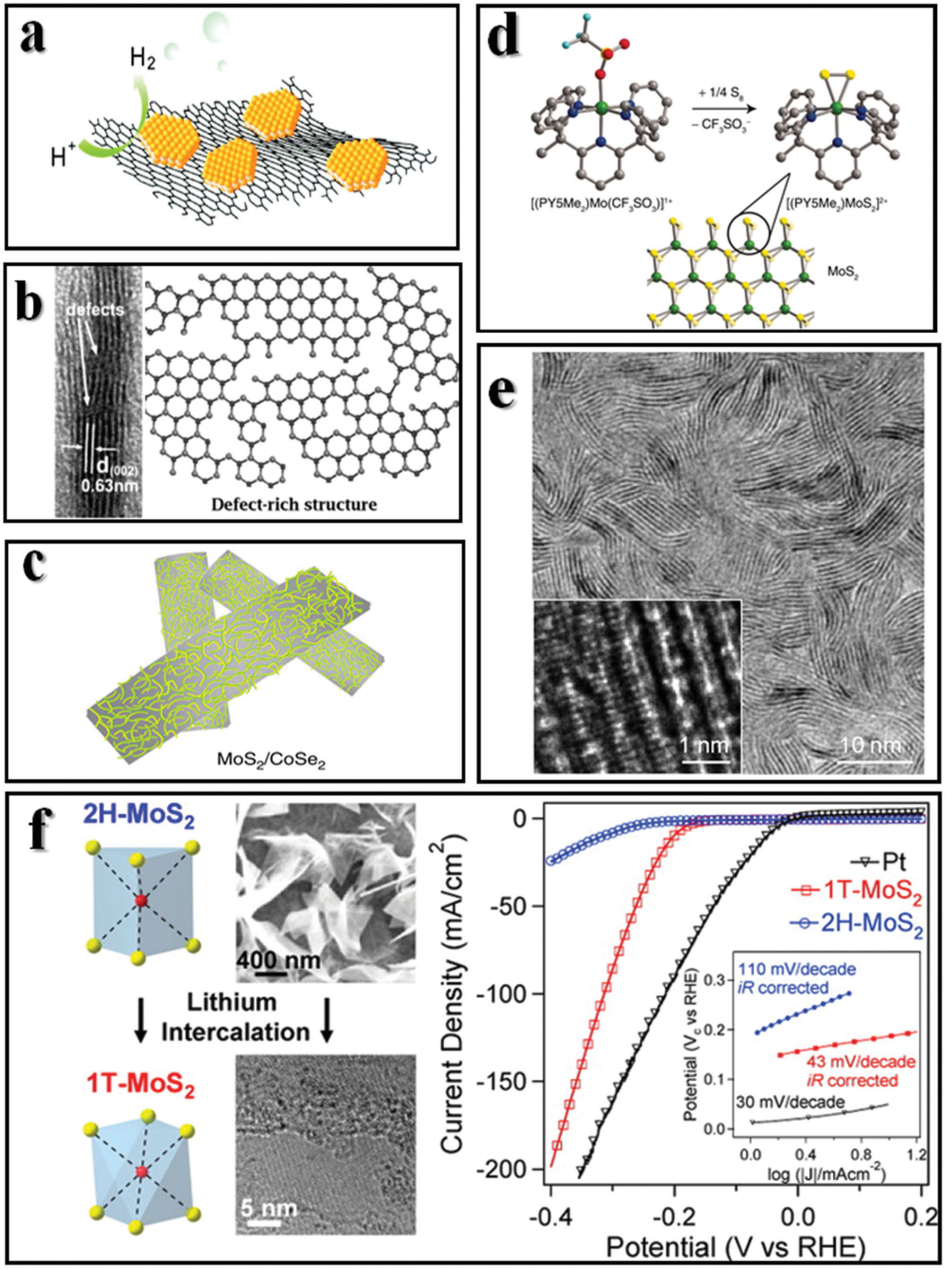
Methods to optimize HER properties. a) MoS_2_ grown on graphene can help to increase the conductivity. Reproduced with permission.^49^ Copyright 2011, American Chemistry Society. b) Defect rich structure can create more edges. Reproduced with permission.^48^ c) Schematic representation of MoS_2_ edges on CoSe_2_ belts, this is the best HER catalyst among non‐noble metal to date. Reproduced with permission.^54^ Copyright 2015, Macmillan Publishers Ltd. d) A molecular mimic of MoS_2_ edges. Reproduced with permission.^46^ Copyright 2012, American Associaton for the Advancement of Science. e) Verticle aligned MoS_2_ structures. Reproduced with permission.^51^ Copyritht 2013, American Chemical Society. f) 1T phase of MoS_2_ can be prepared via lithium intercalation in 2H phase (left), the performances are shown at right. Reproduced with permission.^18^ Copyright 2013, American Chemical Society.

This 1T‐MoS_2_ can dramatically decrease the Tafel slope comparing with 2H‐MoS_2_ in the same acidic environment (**Figure** **2**f).^18^ However, the best way to optimizing activity is not keeping on exploring 1T‐MoS_2_ or some other unstable structures,^44^ but on other practical methods to maintain high stability while keeping accessible activity. There are three basic and comprehensive rules to design more active HER catalysts^45^: increasing the activity of single active site, or increasing the amount of active sites, and the third rule is to increase the conductivity of catalyst. 1T‐MoS_2_ may be activity for HER, but it is not stable, thus greatly hinder the real use. Another way is to design new catalyst with similar active center, since we already know that the active sites are edges and we already know their structures. The research began from the mimic of nitrogenase, and now can go back to small molecule to optimize the active center. A large ligand called PY5Me_2_ [PY5Me_2_ = 2,6‐bis(1,1‐bis(2‐pyridyl)ethyl)pyridine] could generate a new HER catalyst with Mo and S2 dimmer (Figure 2d).^46^ This alternative strategy may open new gate for catalyst design. The second rule seems easier to achieve: Jaramillo group synthesized a highly ordered double‐gyroid MoS_2_ bicontinuous network with nanoscale pores, the high surface curvature exposes abundant edges^47^; Xie group prepared defect‐rich MoS_2_ nanosheets which force the formation of edges (Figure 2b).^48^ As for enhancing the conductivity, Dai group fabricated a strong coupling MoS_2_–graphene sheets, and the conducting network facilitate the catalytic reaction (Figure 2a).^49^

Synthesis of large area thin MoS_2_ is not easy,^50^ fortunately highly active HER catalysts demand vast edges but not large area. Vertical aligned MoS_2_ layers grown on electrode would maximally expose edges as well as increasing the conductivity, although they are not easy to fabricate. The edges are thermodynamically unfavorable with basal plane by 2 orders of magnitude of surface energy, the vertical aligned structure could still be formed through a kinetically controlled rapid growth method (Figure 2e).^51^ In the chemical vapor deposition MoS_2_ synthesis, chemical conversion is a fast step comparing with gas diffusion, if diffusion along the layers through basal plane is much faster than diffusion across layers, then vertical growth is favored. The vertical aligned layers indeed enhanced the activity.^52^ Meanwhile such vertical structures are best platforms to investigate the edge properties, transition metal could dope at edge sites but not on basal planes,^53^ and Li_2_S nanoparticles mainly deposit on the edges versus basal planes in Li‐S batteries test. To date, the best HER catalyst among non‐noble metal is constructed by in situ growth of MoS_2_ edges on CoSe_2_ belt (Figure 2c), which possesses onset potential of ‐11 mV and Tafel slope of 36 mV dec^‐1^, comparable to commercial Pt/C catalyst.^54^

**Figure 3:**
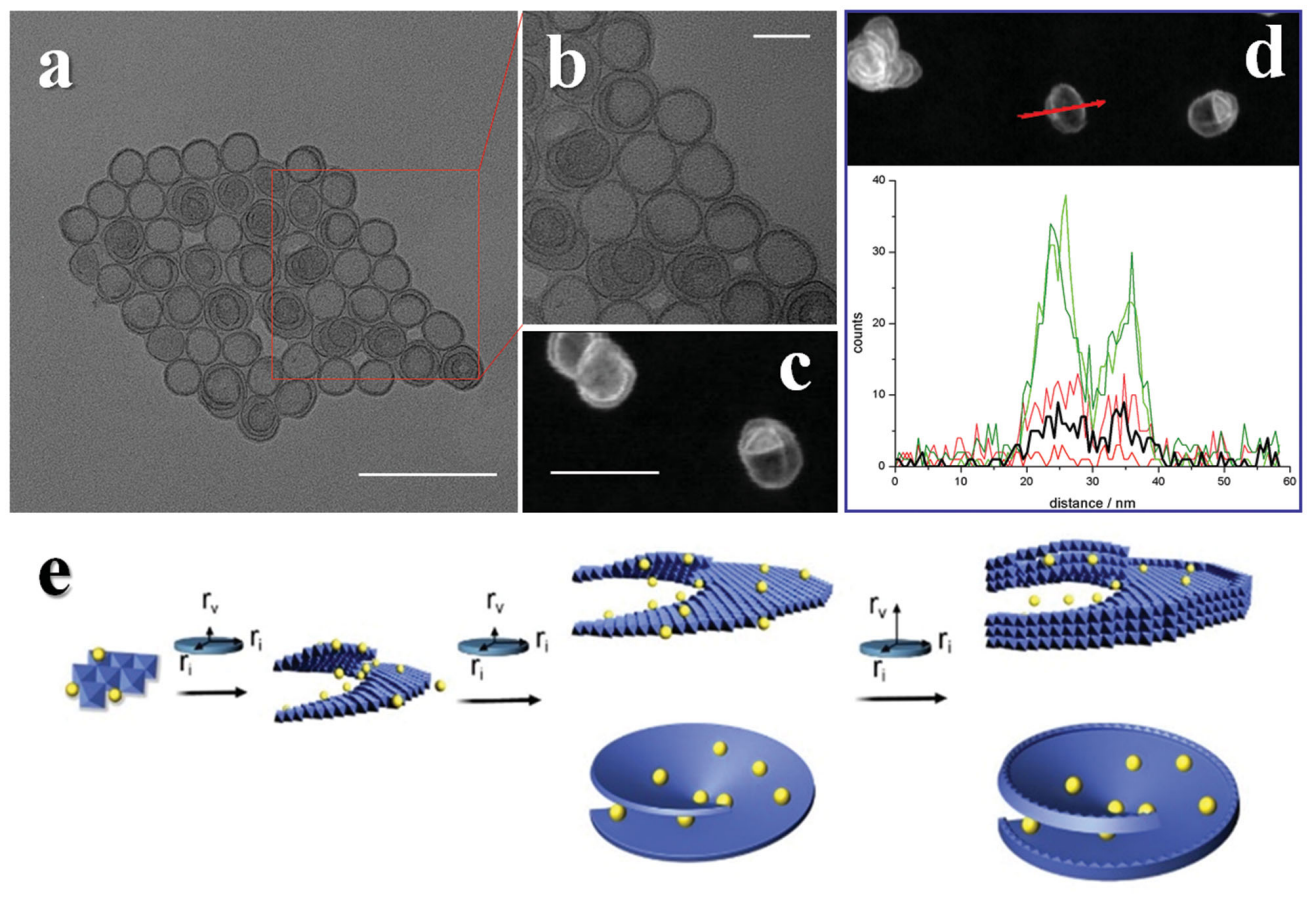
Spiral ultrathin nanosheets with overgrown edges (SUNOE). a,b) TEM and c,d) STEM images of NiFe SUNOE, the element line scan (d, down) indicates the ratios of metal to growth inhibitor vary with position (black line stands for growth inhibitor and other lines stands for metals). e) Growth mechanism of SUNOE, yellow balls represent growth inhibitor, after the concentration of inhibitors in solution decrease a lot, the growth rate of vertical to plane is released and cause an overgrowth at edges. Scale bars: a) 100 nm; b) 20 nm; and c) 50 nm. Reproduced with permission.^55^ Copyright 2015, Royal Society of Chemistry.

Another practical method to decorate edges in layered structures is achieved via edge overgrowth through screw dislocation driven growth^55^ (**Figure** **3**). Crystals can be enlarged through screw dislocations under proper supersaturations in two competing directions, directions of in‐plane and vertical to plane.^56^ For layered structures, the growth rates of these two directions are inherently different, thus the growth manner can be proper controlled. By introducing a growth inhibitor in the growth of layered structure, the growth rate in direction of vertical to plane might be more affected than direction of in‐plane. Then after the depletion of growth inhibitor in reaction media, the growth rate of vertical to plane is released and the edge overgrowth might happen. This is a promising method to sophisticatedly design the edge structures of layered materials. As a proof of concept, ultrathin spiral nanosheets with overgrown edges of NiFe, CoFe and CoNi bimetallic hydroxides are achieved now, and they show promising electrochemical activities for oxygen evolution reaction. The growth mechanism and influencing parameters are easy to accessible, and taking the achievement of several other spiral layered structures^57^, ^58^ into account, the fabrication of more spiral nanosheets with overgrown edges can be foreseen in the future. Since structure determines properties, the properties of such novel structures might be quite interesting.

The edges of MoS_2_ can do more than HER. They can be used in catalyzing hydrodesulfurization,^8^ and methane conversion.^59^ More researches need to be done in designing new structures utilizing the convenience of edge properties and searching new catalytic reactions. Other transition metal dichalogenides (TMD) also experimentally showed promising activities for HER.^60^, ^61^ Meanwhile theoretical study suggests the edge sites of MoSe_2_ and WSe_2_ should be comparable or even better performance than MoS_2_ for HER.^62^ However, the exact edge structures of such compounds under reaction conditions still need to be more explored in order to link with properties.

### 3.2 Metals and CO_2_ Conversion

Au may be the most fascinating metal in history as it can be used as coin metal, mainly because it is inert and scarce on earth. Early experiments using gold as catalyst did not show superior to other catalysts.^63^ But now gold has become a never‐ending frontier in homogeneous or heterogeneous catalyst since the finding of low‐temperature oxidation of CO.^64^ One of the most concerning worldwide problems in the rapidly developing world is the overproduction of the greenhouse gas carbon dioxide led by ever‐increasing worldwide consumption of fossil fuels.^65^ The perfect solution may be conversion of CO_2_ to other useful compounds by using sunlight,^66^, ^67^, ^68^ like what has been done by plants. However efficient catalysts are still in fancy. Finding a feasible method to converse CO_2_ to other carbon forms open a new area for catalyst research. So far, electrochemical reduction of CO_2_ is considered as a promising method and Au is a good platform for mechanism study.

By thermodynamic calculation, the half reactions potential electrochemical reduction of CO_2_ to CO is –0.1 V, but experimentally very negative potential (usually –0.6 V to –1 V) must be applied to initiate CO_2_ reduction. Meanwhile such large negative potential would also trigger the evolution of H_2_ since the half reaction potential to form H_2_ is 0 V. The sluggish in overpotential is chiefly caused by the multi‐step processes of reduction, density function theory (DFT) studies suggests the mechanism include the following steps^69^, ^70^, ^71^, ^72^:

(1) $${\mathrm{CO}}_{2}\;+\;*\;+\;H^{+}(\mathrm{aq})\;+\;e^{-}↔\mathrm{COOH*}$$

(2) $$\mathrm{COOH*}\;+\;H^{+}(\mathrm{aq})\;+\;e^{-}\;↔\;\mathrm{CO*}\;+\;H_{2}O(l)$$

(3) CO* ↔ CO(g) + *

where * denotes a free active site. From these elementary reactions, we can intuitively conclude that materials which could both stabilize COOH* and destabilize CO* would be a prominent catalyst for reduction of CO_2_ to CO.

**Figure 4:**
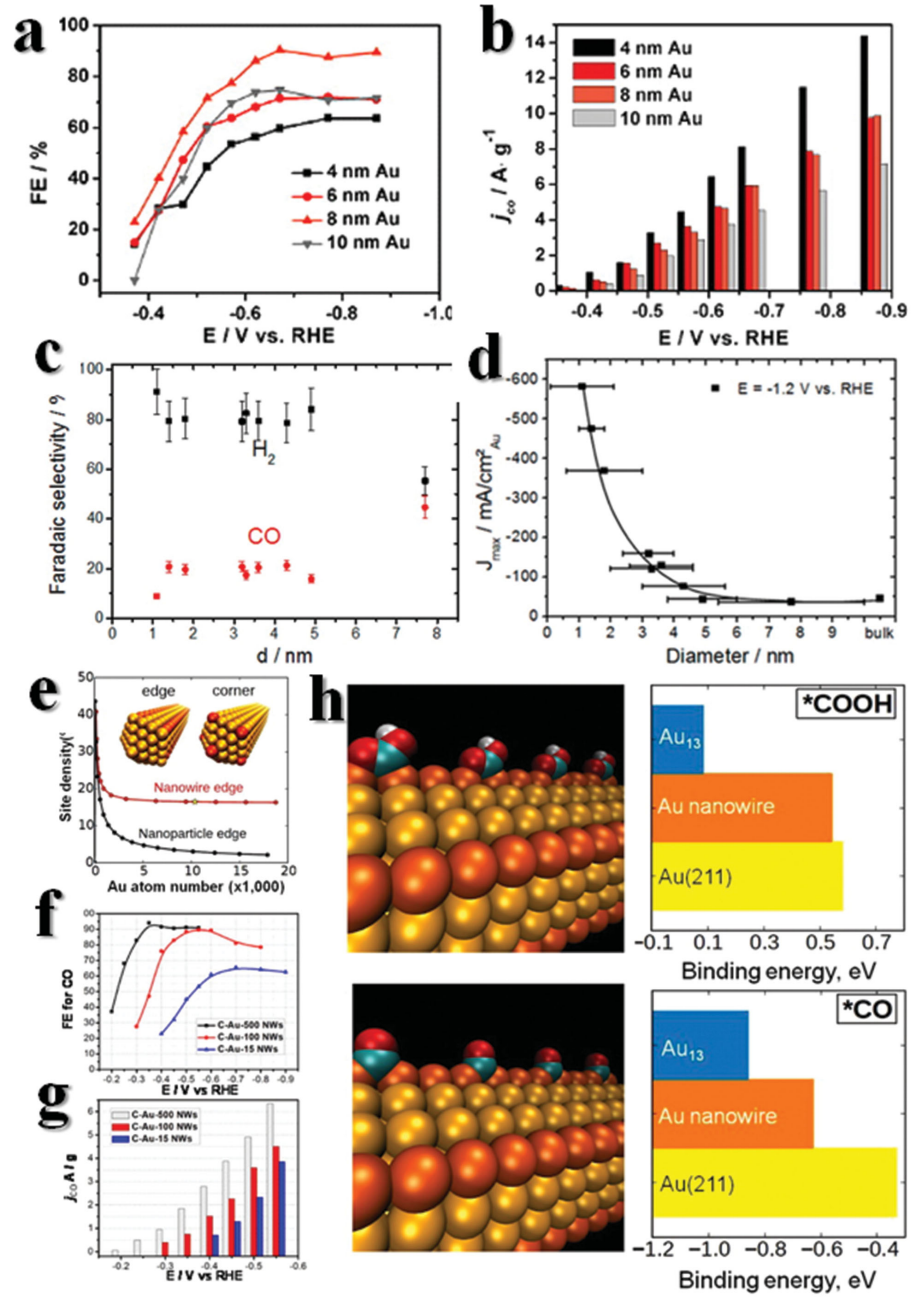
Electrochemical conversion of CO_2_ results of Au nanomaterials. Here show the performances of gold nanoshperes characterized by a,b) TEM and c,d) STM. Data in (c) were acquired at E = −1.2 V vs RHE. Reproduced with permission.^4^, ^74^ Copyright 2013, American Chemical Society. e) While the UNWs get longer, the edge density is much larger than that in nanoparticles. f,g) The property varies with the length of nanowires, the longer the better. C‐Au‐500 (100 or 15) NWs means Au UNWs of 500 (100 or 15) nm length mixed with carbon, respectively. h) Computational results on CO_2_ reduction and CO formation. Configurations of the adsorbed COOH (up) and CO (down) on a Au NW are portrayed here. The edge‐type sites are highlighted in orange. In the Au‐COOH bonding mode, both C and O (carbonyl) bind to Au directly with OH tilting away from the O=C bond. In the Au‐CO bonding mode, one CO binds to two Au atoms with CO serving as the bridge. Binding energies of the key COOH and CO intermediates calculated on the Au NW, Au_13_ cluster, and Au(211) are shown in the right. Reproduced with permission.^75^ Copyright 2013, American Chemical Society.

Many efforts have been made to search high active catalysts and determine active sites in reactions. Small Au_25_ clusters experimentally show much better performance than bulk Au particles, and computational data illustrate the banding energy of CO_2_ with Au cluster vary from sites to sites.^73^ For Au particles with different sizes, the ratios of atoms at edges or surface are different. An optimized ratio is required to reconcile the requirement of maximizing active sites for CO_2_ reduction and minimizing the active sites for HER. Sun group^4^ produced monodisperse Au NPs with different sizes, and found the 8 nm Au particles showed maximum Faradic efficiency (FE) at –0.67 V to convert to CO (**Figure** **4**a,b). The 8 nm Au particles with 4 nm crystallite diameter appeared to meet the demand for optimizing the edge fraction. While Cuenya group found that Au particles below 5 nm all showed significantly better performance than bulk Au, and particles larger than 5 nm were similar with bulk gold (Figure 4c,d).^74^ The disparate activities in these two results may stem from different methods for measurement of size, Sun used transmission electron microscopy (TEM) while Cuenya used atomic force microscope (AFM). However, a similar conclusion made in this two cases is that the edge sites or low coordinate sites are active for reactions, the method to further enhance the activities may be enlarge the fraction of edges in particles or create more edges. This could be achieved by ultrathin nanowires (UNWs), ultrathin structure would maximally expose edges and long nanowire would further elongate the edges (Figure 4e). The ultrathin Au nanowire with diameter of 2.1 nm and length of 500 nm reaches 94% of FE at relative low potential (–0.35 V) (Figure 4f,g),^75^ putting forward the realization of CO_2_ reduction. In computation results, Au(211) is the best for COOH* stabilization, but the binding energy of CO* offset the advantage (Figure 4h). Taking the whole energy effects into account, ultrathin nanowire is a promising candidate for CO formation.

Edges, on one hand, are the active centers, but on the other hand, may be an obstacle for experimental measuring the molecules' binding energy on surface. For commercial metal films, the surfaces are not perfect flat or exposing pure lattice, and there are always terraces and edges in one optical flat surface. However, the adsorption energies are not the same at terraces and edges, and this would lead to disparities for experimentally determining the adsorption energy on facets. Taking CO on Pt(111) as an example, the adsorption energy was found to be 32.3 ± 1 kcal mol^‐1^ by Ertl et al.^76^, but 45 ± 3 kcal mol^‐1^ by King et al.,^77^ and 31 ± 0.5 kcal mol^‐1^ by Campbell.^78^ Bartels group^79^ developed the velocity selected residence time method to remove the effects of different surface structures or defects. The well‐designed technics corroborated that the edges of Pt are special, and bind stronger with CO than Pt(111) facets.

**Figure 5:**
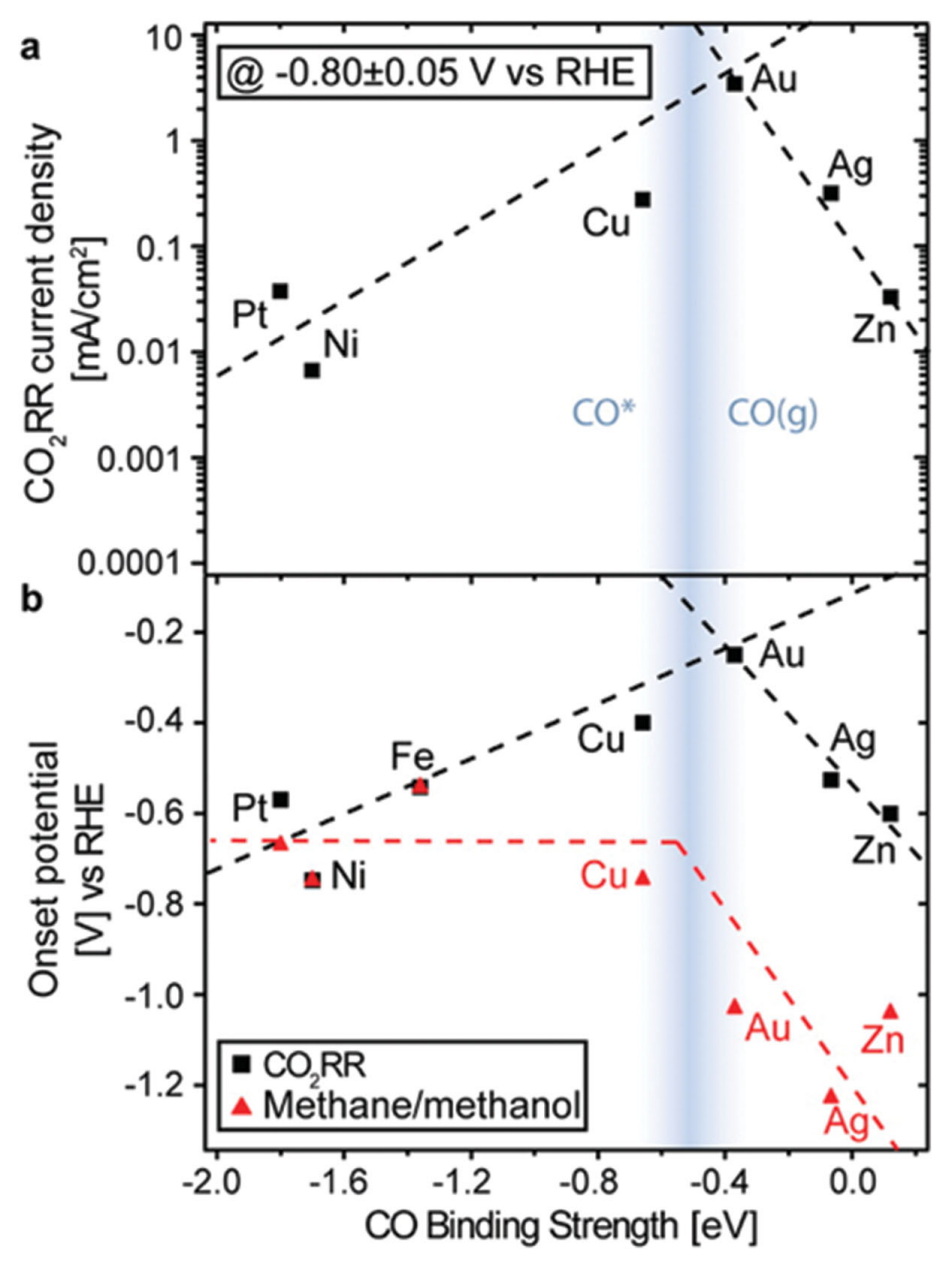
a) Volcano plot of partial current density for CO_2_ reduction reaction (RR) at −0.8 V vs CO binding strength. b) Two distinct onset potentials plotted vs CO binding energy: the overall CO_2_RR, and methane or methanol. Dashed lines are to guide the eye. Reproduced with permission.^80^ Copyright 2014, American Chemical Society.

What if the reactive sites bind CO too tightly? The electrochemical reduction would keep on going and CO_2_ would be further reduced to hydrocarbons. Tests on Au, Ag, Zn, Cu, Ni, Pt, and Fe films all showed the capability to produce methane or methanol.^80^ A volcano plot with CO binding strength as abscissa and CO_2_ reduction current density as vertical coordinates was made by experimental data (**Figure** **5**), suggesting the CO binding is the vital step for CO_2_ conversion. The disparate trends beside the peak may be driven by competition of abilities to stabilize CO* and reduction of CO*.

Copper is a widely studied metal for CO_2_ conversion into hydrocarbon. The fully reduction of CO_2_ requires an eight electron transfer process, thus lots of intermediates and final products may exists in the reaction. A systematic computation study involving 41 different intermediate steps on copper surface illustrated numerous routes may be possible to produce CO, HCOOH, CH_4_ and C_2_H_4_.^70^ Additionally, it is well accepted that Cu(211) facet is more active than (111), (100) facets to generate CH_4_.^71^ One more thing to note is that (211) facet of face center cubic (fcc) packing is not a close packing layer, and expose well‐packed edges of (111) facets. As for the reduction to ethylene, there may be two distinct mechanisms: one is through the formation of a surface‐adsorbed CO dimer at (100) facet at relatively low overpotentials,^81^ and the other has a methane intermediate at both (111) and (100) facets.^82^ To further corroborate the mechanism in real condition, in situ study is required.^83^ Since CO_2_ conversion is a multistep process, we can design multicomponent catalysts to work cooperatively to stabilize or destabilize some intermediates, and lead the reaction to reach a single products.^84^ One advantage of NPs to serve as catalysts is that the amount of atoms on surface is enlarged, meanwhile the edge fraction is also increased. The 7.0 nm Cu NPs reach up to 4 times methanation current densities comparing with high purity copper foil electrodes.^85^ However, they are not stable under electric treatment. Alloying Cu nanoparticles with Au would enhance the stability, however, it would decrease the selectivity.^86^

### 3.3 Reactions at Edges of a Multicomponent Interface

As we can see from previous discussion, a catalytic reaction usually requires the stabilization of some intermediates and destabilization of some other intermediates. Such observation enlightens us to pay attention to multicomponent interface, especially the edge of the interface. Elaborately selecting various active compounds and carefully tailoring the combination of multi components would realize lots of unexpected performance. This trial has started long time ago, Tauster et al. found strong metal‐support interactions (SMSI) in TiO_2_‐support noble metals, and the CO and H_2_ absorption capacities was lost at high temperature.^87^ The SMSI effects are detrimental in many cases,^88^, ^89^ thus researchers would like to avoid them and choose inert supports in early studies. Recently, more and more examples^16^, ^22^, ^23^, ^90^, ^91^ proved that the supports might promote some reactions and enhance the total catalytic activities.

**Figure 6:**
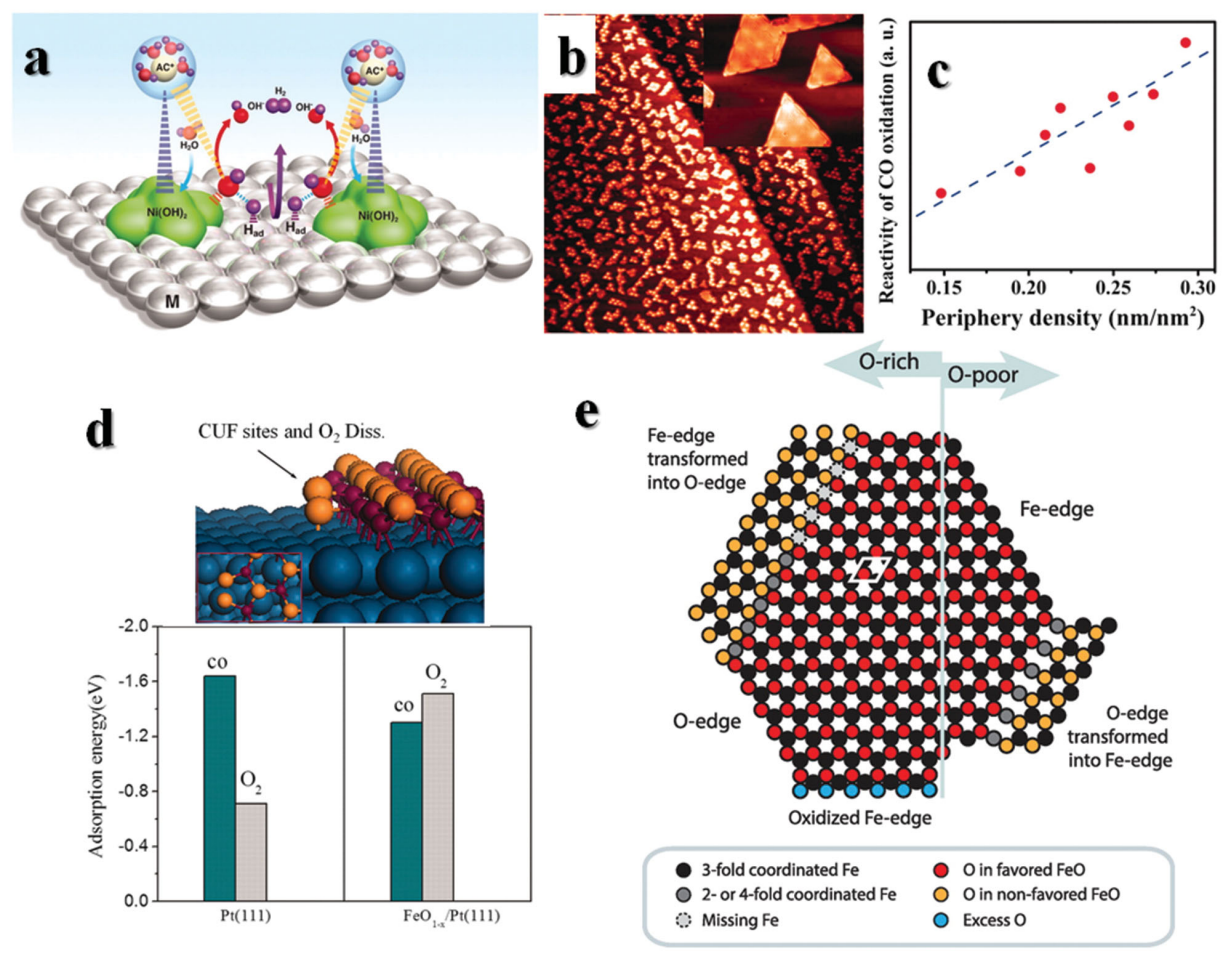
a) Schematic illustration of the cooparation of Pt and Ni(OH)_2_ to enhance HER properties. Reproduced with permission.^92^ Copyright 2011, American Association for the Advancement of Science. b) STM image (200 nm × 200 nm) from the FeO_1‐*x*_/Pt(111) surface. Inset is an atomic‐resolution STM image of FeO monolayer nanoislands (25 nm × 20.8 nm) recorded at liquid N_2_ temperature. c) Dependence of reactivity of CO oxidation on the periphery density (edge density). d) Schematic structure of the Coordinatively unsaturated ferrous (CUF) sites and calculated transition states of O_2_ dissociation (the inset shows the top view) at the boundary between FeO and Pt(111). The calculated adsorption energies (in eV) for CO and O_2_ molecules on Pt(111) and FeO_1‐x_/Pt(111) surfaces are different. Reproduced with permission.^94^ Copyright 2010, American Association for the Advancement of Science. e) There can be found five types of FeO_1‐x_/Pt edge structures. Here is a brief summary of them. Reproduced with permission.^24^ Copyright 2015, American Chemical Society.

Taking HER as an example, metal like Pt can easily adsorb atomic hydrogen and subsequent form H_2_, so the last thing for HER catalyst design is choosing a proper compound to dissociate water and coupling these two materials in a proper manner. Ni(OH)_2_ is a good candidate for such demand. A controlled arrangement of Ni(OH)_2_ clusters on Pt(111) (**Figure** **6**a) can enhance the activity by a factor of 8 compared to Pt.^92^ The active sites sit on the well‐arranged edges of Ni(OH)_2_, which remind us to think about multi active centers in homogeneous reactions. In both areas, one compound plays only a part in the catalytic reaction by its own advantages, and multi‐compounds are precisely placed in order to achieve the well communication of intermediate steps. The difference of them are their complete structures, and homogeneous catalysts with multiple active centers usually have elegant molecule constructions with certain constitutions, while in heterogeneous catalysts, they only have well‐controlled active centers.

Composite structures with transition metal oxides on noble metals are called inverse catalyst,^93^ in order to distinguish them from conventional metal on support catalyst. A well‐studied case of inverse catalyst is ferrous oxides on Pt substrates (Figure 6b).^94^ Pt catalyst is easily poisoned by CO because of the strong adsorption by an adsorption energy of –1.64 eV on (111). Such a strong interaction would block all the surface Pt atoms and prevent other substrate adsorption, thus no other reactions would happen on Pt(111) in the present of CO. However, on the edges of FeO_1‐*x*_ monolayer islands, the binding energies change a lot (Figure 6d), and preferentially adsorb and activate O_2_. Then spontaneous oxidation of CO would validate even at room temperature, depending on the periphery density or edge density of FeO_1‐*x*_ islands (Figure 6c). Fully covering the Pt(111) with FeO_1‐*x*_ exhibits minimal reactivity at room temperature due to the lack of edges of interface.^93^ NiO‐on‐Pt^95^, ^96^ and CoO‐on‐Pt^97^ can also be obtained in similar methods. Among this three catalysts, FeO_1‐*x*_/Pt(111) shows the best performance of preferential oxidation reaction of CO in the presence of H_2_ (PROX), while NiO_1‐*x*_/Pt(111) is best for oxidation of CO in the absent of H_2_ (COOX).

The reaction demands elaborate collaboration between two functional compounds on the edges of one. However, ionic or metallic bonds are not similar with covalent bonds. They cannot be fixed into a certain direction, and defects or distortions cannot usually be avoided in real materials. There have been found 5 different edges structures of FeO monolayer islands on Pt(111) (Figure 6e),^24^ depending on the synthesis environments.^98^ Computational^99^, ^100^ or experimental^101^, ^102^, ^103^ works have shown activities with different O‐edges or Fe‐edges are distinct. Meanwhile, oxygen vacancies near edge would also affect the oxidation mechanisms, “spectator” mechanism or “carboxyl” mechanism of reduction to C2 compounds are possible depending on edge structures.^104^ Besides FeO monolayer islands on Pt(111), CeO_*x*_ and TiO_*x*_ on Au(111),^105^ FeO on Ru(0001),^106^ CoO on Ir(100)^107^ and NiO on Rh(111)^108^ have also been synthesized and studied in various situations, more comprehensive studies could be find in reviews by Freund et al.^109^ and Surnev et al.^110^

In such inverse catalysts, the metal oxides are usually composed of monolayer or several layers, the active sites reside on the edges of metal oxides. The multicomponent interface may owe their origin of activities to the “coordinate unsaturated”.^93^ In other cases, there are many beneficial SMSI effects on metal oxides support metals,^23^, ^111^ and sometimes the enhanced performance cannot simply derive from “coordinate unsaturated”. When using reductive metal oxides as supports, the reducing nature of supports would whether donate or withdraw charges from metals near the metal/oxide interface, depending on the work functions of two materials.^22^ SMSI effects cannot be simply concluded. The Pt NPs on iron oxide may undergo single layer encapsulation with Fe migration driven by SMSI.^112^ This effect is similar with under potential deposition in electrochemistry.^113^ To some extends, we may consider such interface as a new chemical compound, since it possesses new characters comparing with each single materials.

**Figure 7:**
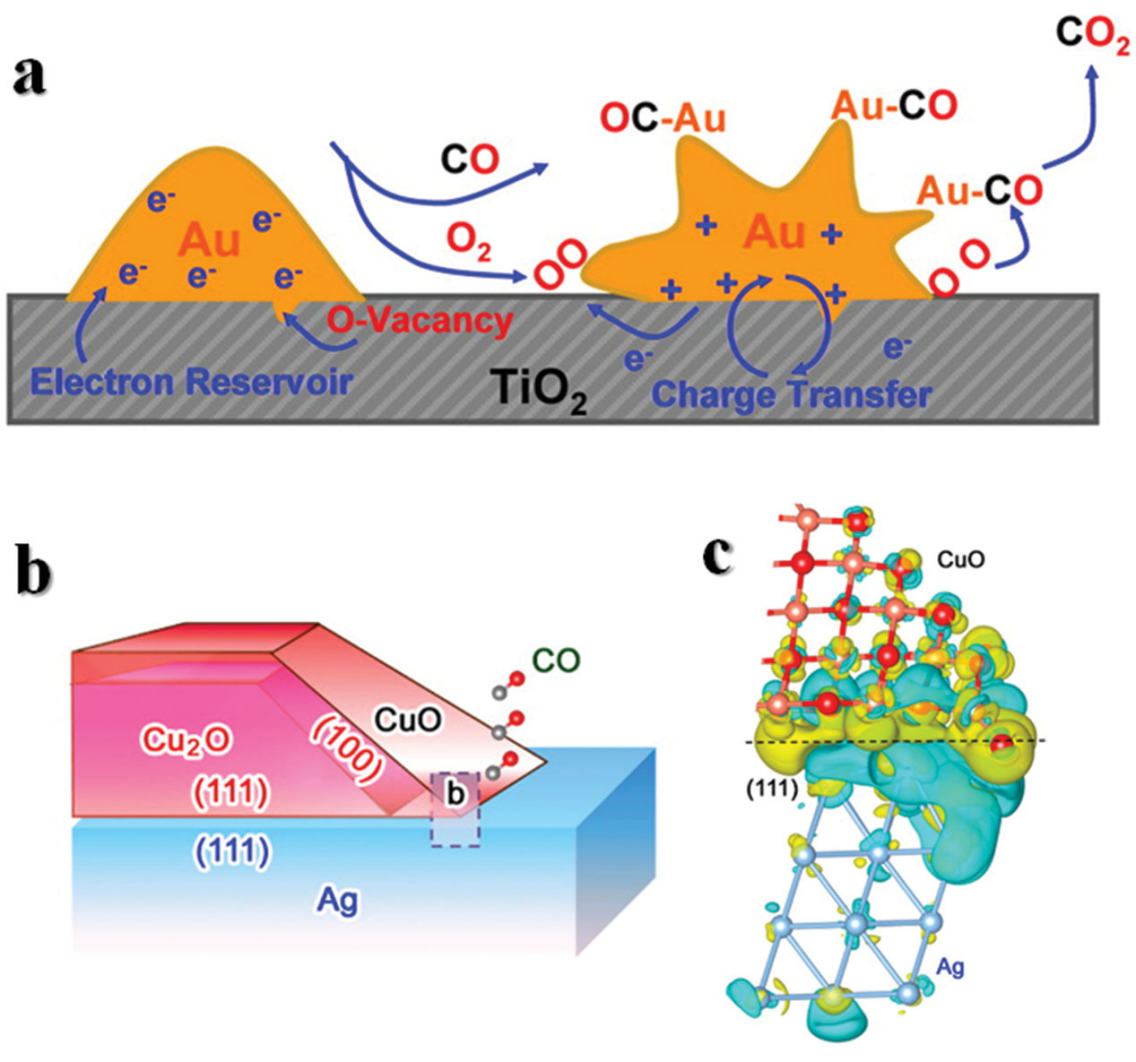
a) Schematic illustration of Au_16_ on TiO_2_ (110) for CO oxidation involved the generation of oxygen vacancies. Reproduced with permission.^22^ Copyright 2013, American Chemical Society. b) Schematic illustration for the Ag‐CuO/Cu_2_O hybrid structures. c) Differential charge density by first‐principles simulations illustrates the increase (olive color) and decrease (cyan color) of electron distributions. Reproduced with permission.^114^ Copyright 2014, American Chemical Society.

In a hybrid structure of CuO covered Ag interface, the CO oxidation activities is linked with the interfacial lengths.^114^ Ag is inert for CO oxidation, while CuO can oxidize CO to CO_2_ with an activation energy of about 62.8 kJ mol^–1^. However, the partial covered structures possess apparent activation energies range from 36.7 to 42.7 kJ mol^‐1^. DFT calculations reveal that charge redistributes in the interface (**Figure** **7**b), the charge is enriched at CuO surface near interface (or edge of CuO), while for space far away from interface, the charge density almost keeps same. In many cases, the interactions are more complicate, the adhesion of gold on TiO_2_(110) differs with different treatments of TiO_2_,^115^ dual catalytic sites in the composite are possible for CO oxidation,^116^ Au_16_ on TiO_2_ (110) for CO oxidation involves the generation of oxygen vacancies (Figure 7a).^90^ In another case, a simple synergistic effect of different active compounds without charge redistribution may be essential for selective catalytic oxidative dehydrogenation of carboxylic acids.^117^ The study of SMSI or interface need to be more studied, then more rational rules can be drawn.

## 4 Novel Structures and Atypical Edges

Vast morphologies of nanomaterials from 0D to 3D and their composites have been achieved in the past several decades.^30^, ^91^, ^118^, ^119^, ^120^ Among them, novel structures like nanoframe (NF) and ultrathin nanowire (UNW) have attracted lots of attention both in nanostructure fabrication and applications. NFs are NPs with interior atoms etched out and atoms on edges preserved. From the sight of pristine NPs, the NFs are absolutely the edges of them, and the research on NFs represents the study of edges of pristine NPs. However, if we consider NFs as newly formed NPs, the edges of them are difficult to define, the structures of NFs are not easy to detailing. For convenience, we consider the whole NFs as atypical edges. UNW is a kind of unique one dimensional material. The atomic structures of UNW are, in many cases, not quite clear yet, and thus it would be difficult to define the edge of UNW, or determine whether the UNW as an entirety could resemble edge. From the sight of geometry, both UNW and conventional edges are one dimensional structures, and they may both feature low CN, too. So it might be proper to consider the UNW as edges. A brief comparison between these two novel structures, which could be seen as atypical edges, with conventional edges is made in **Table** **2**, the specified discussions of them are made in the following parts.

**Table 2:** A brief comparison between conventional edges and two kinds of atypical edges

	Conventional edges of nanostructures	Nanoframe	Ultrathin nanowire
Structural feature	Natural ends of surfaces in nanostructures	Structures with internal atoms on surfaces and in body of particles etched out and atoms on edges reserved	Ultrathin one dimensional materials
Chemical feature		Low CN and large edge content	Low CN (maybe)
Advantage	Easy accessible and abundant types	High activity and low use of costly materials	Enhanced activity
Limits		Difficulty in fabrication; Not all NFs are stable	Dimness in detailing structures

### 4.1 Nanoframes and their Unique Properties

Reaction on metal surface depends on the nanoscale structure of metal.^29^ For atoms on metal surface, the CN changes from corners, edges to surfaces. Lower CN is always related to high active center. Thus a stable structure with numerous low CN sites is widely pursued in research and industry. Corner sites seem perfect but impractical because of the low content. Stubbornly reducing the size would risk the stability, and lose the merits of nanomaterials – easy recycle. Meanwhile the internal atoms have few chances to contact with substrate. Decreasing the amount of such unnecessary atoms would enhance the mass activity and reduce the employment of scarce metals. Then a structure with pure edges seem like a promising solution for reducing the amount of catalysts while maintain or enhance the activities. Thanks to the great development in nanomaterials synthesis, innumerable morphologies have been prepared in the past several decades, especially in the last 15 years. NFs with internal atoms etched out and edges conserved can be realized in many cases. These unique structures are ideal platform for edge studies comparing to integrated solid particles, because of simplified interference. However, the catalytic performance study is still in fancy because the challenge in synthesis is still the first obstacle for systematically research. Here we briefly introduce the fabrications of NFs, and some of their enhanced performances in catalytic reactions. A more comprehensive summary could be made only after vast researches have been carried out.

#### 4.1.1 Conventional Nanoframe Synthesis Methods

From the concept of thermodynamic, NFs are not stable, like nanocrystal with high index facet.^121^ Fortunately, lots of crystals with high index facets have been generated by dynamic control, so have been in NF synthesis. There are lots of similarities both in synthesis and properties in these two areas, in brief, there are lots of atoms with low CN in both two cases. When we talk about edges in daily life, we usually refer to the ends of a flat surface. However, there is no flat surface in atom scale if we consider the atom as a sphere. What makes the edges of NPs so special is mainly the low CN. We may define the edges in nanoscale by CN changes in facets, because the CN is always lower at edges comparing with facet atoms. Here we consider a face center cubic package as an example (**Table** **3**). For cubic structure enclosed by (100) facets, the nearest CN of atoms on (100) facet is 8, edge is 5 and corner is 3. While as for tetrahedron with only (111) facets encased, the nearest CN of atoms on surface is 9, edge is 6 and corner is 4. For (111) facets enclosed octahedron, the nearest CN of atoms on surface is 9, edge is 7 and corner is 3. It seems ok to define the edges as the atoms possess the second lowest CN on the surface. However, there would be great confusion in particles enclosed by high index facets. For instance, there are two kinds of atoms on (211) facets, one has nearest CN of 7, the other has 9, meanwhile edge is 7. Nearest CN of a series of atoms on surface could reach the nearest CN of edges of octahedron, and is the same with the edges of (211) facets. However, they are not atoms on the same environment because their CN of longer distance are different. Actually crystals with high index facets also represent high activity in many cases,^118^, ^122^, ^123^, ^124^, ^125^, ^126^ as well as edges. For accuracy and simplicity, we consider the edges as the turn of facets. Thus NFs could be seen as pure edges without other surface or interior atoms.

**Table 3:** CN of nearest atoms in face center cubic packing particles enclosed by different facets

	Cube (100)	Tetrahedron (111)	Octahedron (111)	High index facet (211)
Facet	8	9	9	7 or 9
Edge	5	6	7	7
Corner	3	4	3	

To get a NF constituted by edges, an etching process is always required. There are two kinds of typical conventional synthesis methods based on the etching methods. One is a template assist etching process, metal is firstly deposited on the edges of a well‐controlled solid template particle, then a proper etchant is introduced and the template is removed, leaving the frame alone. The other method is a kinetic controlled dissolution process, a stable or metastable particle is generate at first, then another etchant or nothing is added to move out the internal atoms, depending on the stabilities of NPs. Both of these two process are driven by kinetic control, the former one requires two motif to constitute an integral particles, the growth of frame on particle is controlled by kinetic effects. While the etching process of second method is controlled by kinetic effects. We briefly take a view at this two kinds of methods in the following sections.

*Template Assist Etching Process*: Seed mediated growth is powerful to prepare core‐shell structure. Then how to deposit the second material only on the template's edges in order to get frame structure? The solution relies on two aspects: surface chemistry and specific affinity on edges. Nanomaterials are usually fabricated in the presence of capping agents. The introduction of capping agents adsorbed on surface is to inhibit the further enlarge of the particles. Modifying the surface chemistry of nanoparticles is the first consideration of nanomaterial applications.^127^ By selecting proper capping to cap the facets, we can deposit other wanted materials on the active edges and corners. Subsequently etching the template would lead to a frame portraying the outline of the pristine NP. As for specific affinity on edges, this is usually realized in Au‐Ag system,^128^ they are easy to react in suitable position under controllable conditions. The specific affinity also exists in some other nonmetallic materials.^129^

**Figure 8:**
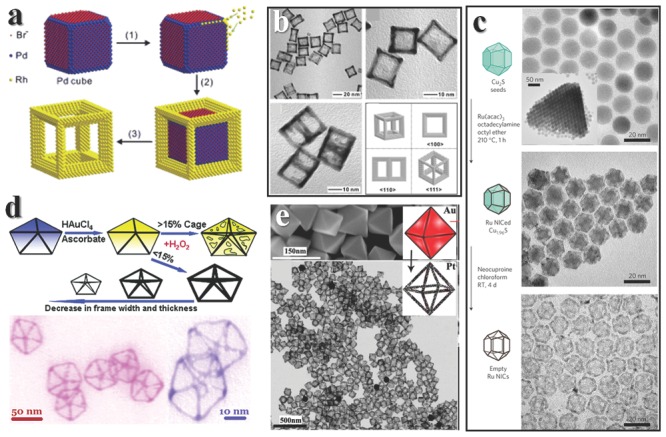
The growth of NFs. a) Formation of Rh NFs with the help of Pd cube. b) TEM images of Rh NFs. Reproduced with permission.^130^ c) Ru NFs can be fabricated via growth on edges of Cu_2_S seeds and then the etching of seeds. Reproduced with permission.^135^ Copyright 2010, Macmillan Publishers Ltd. d) Au atoms can deposit on the edges of Ag decahedra, thus lead to the formation of NFs. Reproduced with permission.^134^ Copyright 2011, American Chemical Society. e) Pt can grow on the edges of Au (up) and form NFs (down). Reproduced with permission.^128^ Copyright 2012, American Chemical Society.

We first look at an instance of surface chemistry controlled NF fabrication. Xia group successfully synthesized Rh cubic NF by etching the Pd template (**Figure** **8**a).^130^ They firstly synthesized Pd cube enclosed by Br^−^ capped (100) facets, then reduced Rh salts at the edges of Pd cube. The Rh would selectively grow on the edges because of the passivated (100) facets, and preferentially grew along <111> direction at optimized precursors concentration because of a kinetic effect.^122^ When Br^−^ was absent in the system, Rh could also grow on the surface. Due to the inherent different resistance to oxidative corrosion, Pd cube could be etched and Rh frame could be obtained (Figure 8b). In fact kinetic control here is vital, too, however, the surface control is the starting step of the heterogeneous growth of Rh, thus we categorize it into template assist etching process.

We then take a view at NF fabrication driven by specific affinity on edges. Ag and Au are elements in the same group, their atom radius, lattice structures and parameters are quite similar, thus behave similar behaviors in many cases. However, the reduction potential of AuCl_4_^−^/Au is 0.99 V vs SHE, and AgCl/Ag is 0.22 V vs SHE, thus AuCl_4_^−^ can etch Ag spontaneous, one AuCl_4_^−^ can react with three Ag atoms. The surface energies of different facets usually increase in the order γ(111) < γ(100) < γ(110).^131^ It is reasonable to hold the point that Au would preferentially etch (110) facets, then (100) facets, and the last are (111) facets. Au triangle frames can be generated by galvanic etching Ag triangular nanoprisms enclosed by (111) and (110) facets.^132^ The Au indeed etches Ag atoms on (110) facets and depose on (110) facets. If the etching is achieved only by AuCl_4_^−^, the thickness and roughness cannot be well controlled. However, introducing the second etchant at proper time would be salutary for morphology control, because of the sudden depletion of Ag. Au triangle frames with thickness less than 2 nm or 6 nm could be synthesized in such way.^133^

There are several kinds of NPs that encased only by (111) facets. When using Ag decahedra as template, Au atoms would preferentially deposit on edge sites because of low CN. After H_2_O_2_ etches the Ag decahedra, an Au decahedral NF is generated.^134^ Controlling the amount of Au loaded would help to prepare frames with different thickness. While the gold deposition on (100) facets may lead to a porous mesh in similar reaction procedure (Figure 7d). Pt NFs could be generated by using Au as seeds, and the principles are similar with the Ag‐Au systems. When the edge sites of decahedra Au erode because of low CN, new facets are produced and Pt are preferentially deposited on the newly formed faces. After etching and annealing, smooth Pt NFs can be obtained (Figure 8e).^128^ Pt octahedral frames can be fabricated by using Au octahedron as templates.

Metal NPs are not the only choice for templates. When using Cu_2_S as template to grow Ru, hexagonal biprism Ru NF can be synthesized after etching the Cu_2_S (Figure 8c).^135^ This is a case of edge growth with no specific affinity between templates and metal, the details of driving force still need to be explored. Saturated NaCl aqueous solution can be used as cubic template, too. Adding AgNO_3_ into the solution would directly generate NaCl/AgCl core‐frame structure, then using water to wash the products would take the NaCl core away and leave a frame of cube. Soft reduction treatment would lead to the formation of Ag@AgCl NFs.^136^

The template assist etching process is benefit for structure well‐control. There are many tunable parameters to get ultrathin and ultrafine structure. The combinations of templates and frames are versatile and easy accessible. However, the common rules in this method is not well established yet. The design of new frames need to be ingeniously designed, and lots of trial‐error experiments are required. There is still plenty room in this areas. Well‐controlled synthesis of NFs would certainly lead to new insights both in material fabrications and catalytic processes.

*Kinetic Controlled Dissolution Process*: When something is not thermodynamically stable, an efficient procedure to achieve it is by kinetic control. All practical chemical reactions are less or more away from ideal thermodynamics. Kinetic effects even dominate in vast real processes. In the aforementioned template assist etching process, the deposition of frame material on template edges is usually controlled by kinetic effects. However, the specific character of such process is the present of template, so we name it by template. Herein we discuss kinetic controlled dissolution process with no template involved, the only driving force may be kinetic effects. There are also two forms in this process, based on the stability of the intermediates. In one case, metastable particles as intermediates are formed at beginning because of a fast reaction, after dissolution of internal atoms in the present of low concentration oxidant, finally frames are formed. In the other case, a stable particle is generate as the intermediates, then proper etchant is introduced. Based on the inherent difference of edges and surfaces, something would remain. If edges remain, NF is fabricated. If edges dissolve, the trial fails. Etching the nanosphere would lead to hollow structure or yolk‐shell structure because of the different migration rate, this is so called Kirkendall effect.^137^ Regular NFs would be formed under suitable environment driving by Kirkendall effect.

**Figure 9:**
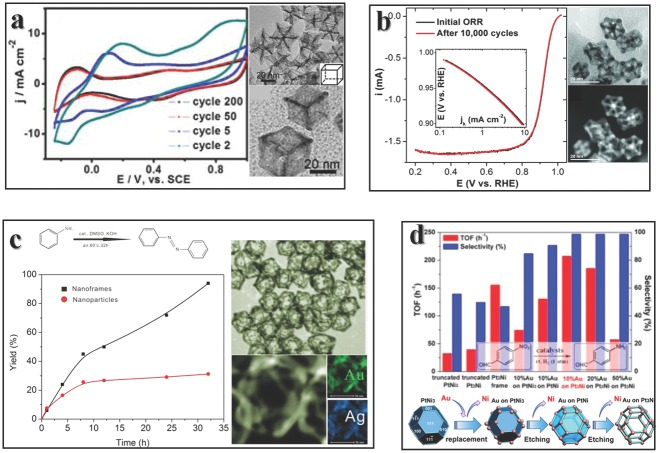
Enhanced properties of NFs. a) CV of PtCu_3_ indicates a degration of NFs. Reproduced with permission.^141^ Copyright 2012, American Chemical Society. b) However, the Pt_3_Ni NFs are quite stable. Reproduced with permission.^138^ Copyright 2014, American Association for the Advancement of Science. c,d) NFs could serve as catalysts for organic synthesis. Reproduced with premission.^140^, ^143^ Copyright 2012, 2014, American Chemical Society. The TEM images inset in panels (a,b,c) are the pristine NF structures, and the cartoon image at the bottom in panel (d) depicts the fabrication method of Au on the corner of NF.

PtNi_3_ polyhedra is not a thermodynamic stable particle because of the active Ni atoms on surface. After the synthesis of PtNi_3_ NP, Ni atom on surface would easily leak out because of the existence of dissolved O_2_ in solvent even at room temperature. Finally the Pt_3_Ni NFs are generated in the solution (**Figure** **9**b).^138^ Pt_4_Ni NFs could be prepared via similar process.^139^ DFT calculations illustrate that after Ni atoms dissolved, Pt atoms on the surface aggregate to form a “porous Pt shell”. Then leaking the internal Ni atoms dominates the dissolution driving by Kirkendall effect and results in the NFs.^140^ PtCu_3_ NFs would be fabricated in similar way.^141^ However, the details still need to be further explored because similar PtCu_3_ can be generated in quite different conditions (Figure 9a).^142^ Ag‐Au octahedra NFs could be achieved in a two‐step process.^143^ Ag nanosphere is prepared at the beginning of the reaction, then galvanic etching by AuCl_4_^−^ could change the morphologies and compositions, and finally Ag‐Au NFs are produced (Figure 9c). Such alloying‐dealloying process could finely control the final morphologies, comparing to the direct etching process to get NFs, though both of them may form NFs as final products under suitable conditions.^144^, ^145^, ^146^

We have discussed the metal NF so far, oxides or other materials of NFs have also been synthesized by etching corresponding materials. Cu_2_O NFs can be synthesized by a rapidly etching process.^147^ Details studies reveal that the (100) faces of Cu_2_O are stable under HCl etching,^148^ thus different novel structures can be fabricate by using various substrate. However, the (100) faces are less stable in other oxidative etching conditions, and leave NFs with part of other facets remained.^149^ Ag_2_O NFs can be formed in a similar manner with Cu_2_O frames,^150^ the stability under etching is in the order (111) > (110) > (100). Taking advantages of this rule, various NFs structure is realized from diverse substrates.

Yet we have seen lots of NF synthesis, however the fabrication methods are still limited in several compounds. General and practical methods is still in fancy. The past several years have witnessed great success in material synthesis and applications, new materials hit headline in research almost every day. Thus, we are confident to predict that NFs would earn their status in the soon coming future.

#### 4.1.2 Applications of Nanoframes

Due to the limitation in synthesis, only a few researches have focused on their chemical advantages, while some of the researches make use of their novel structures or physical properties to realize new phenomena. For example, Xia group capsuled the gold nanocages with thermal response polymer, then light induced phototermal effect of gold can manipulate the cages to open or close to release the chemicals inside.^151^ There are also many studies concerning the surface‐enhanced Raman spectroscopy (SERS) effects of Ag^152^ or Au^153^ NFs or their assemble structures.^154^ Meanwhile, the cage structure can also store small NPs inside and prepare new morphologies.^135^ There are also some researches mentioned the enhanced catalytic performances of NFs, we present some researches at this area in this section.

As we have mentioned in the previous discussion, the NF structures are not thermodynamically stable, especially for the alloy. This is indeed true in Pt‐Cu NF.^141^, ^142^ As depicted in Figure 9a, the pristine cyclic voltammogram (CV) curve shows no under potential H ad/desorption peaks (‐0.24–0.1V), due to the Cu coverage on the surface. As the bias potential applied, the intensity of Cu dissolution peak decreases further, and finally the shape of CV curve resembles to commercial Pt CV curve, meanwhile, shows better performance comparing to PtCu_3_ nanoparticles. The electrochemical dealloying has been proven to successfully synthesize highly active electrocatalysts.^155^ In another test, the Pt‐Cu NFs show higher performance than commercial Pt catalyst.

NFs are not always fragile in any cases. The aforementioned Pt_3_Ni NFs^138^ could maintain their morphologies and activities even after 10 000 cycles without loss for oxygen reduction reaction, while stay a factor of 22 enhancement in specific activity and 36 in mass activity (Figure 9b), which is essential for proton exchange membrane fuel cell.^156^ The secret of stability may be ascribed to the Pt‐skin coverage on the surface, preventing the aggregate of oxygenated intermediates. This hint enlightens stable NF design: cover the frame with something active but stable. Pt‐Ni NFs could also serve as hydrogenation catalyst.^140^ Considering the hydrogenation of 4‐nitrobenzaldehyde as a model reaction, different catalysts were tested. As drawn in Figure 8d, Pt_3_Ni NF shows high selectivity while the turn over frequency (TOF) is relatively low comparing with other truncated structure. A good way to enhance both the activity and selectivity is by sophisticated loading Au on the frames. A proper load content would help to both increase selectivity and activity (Figure 9d).

Several other reactions are employed in illustrating the enhanced performances. Au triangular NF with tailorable thickness range from 1.8 nm to 6 nm shows about 2.4 times reduction rate of 4‐nitrophenol, comparing to frames with thickness larger than 10 nm.^133^ In catalytic reaction, the Au NFs show a reaction rate about 2 times faster than Au nanoboxes, and much faster than solid particles.^157^ Oxidizing aniline to form aromatic azo compounds under mild condition could be achieved by Au‐Ag octahedral NFs, the yield is about 3 times higher than Au‐Ag NPs at 32 h (Figure 9c).^143^ The applications of NFs are not restricted in organic synthesis. The Ag@AgCl NF could degrade methyl orange under irradiation and show great efficient comparing to P25.^136^ A Cu_2_O‐Au complex NF was test to degrade methylene blue and results shown faster degradation than Cu_2_O.^158^

In summary, NFs have shed light on improving performances of catalysts. However, there are still many problems need to be solved besides obstacles in synthesis. Stability under reaction is the first concern for practical application. Though NFs possess innate instabilities, there are ways to avoid it while maintain high activities. The second thing lies on the way is that easy to scale up. Low harvest is accessible in lab, but unacceptable for industry. Exploring new reactions would also benefit for NFs applications.

### 4.2 Ultrathin Nanowires Resemble Edges?

In the view of quantum theory, size determines the boundary condition of wave functions of material. If “the underlying physical laws necessary for … the whole of chemistry are thus completely know”,^159^ then size would no doubt determine the structures and properties of materials. From the points of geometry, the content of atoms at edge becomes larger and larger when the particle get smaller and smaller. However, as we described before, stubbornly reducing the size would risk the stability, and lose the merits of nanomaterials – easy recycle, NFs could be a solution. On the other hand, UNW may be another solution, as we have seen in section 3.2, ultrathin Au nanowires which possess high edge atom content could improve the CO production.^75^ Ultrathin Pt nanowire with diameter about 3 nm also shows enhanced properties of electrochemical oxidation of formic acid or methanol comparing with commercial Pt/C catalysts.^119^ The results show promising future for application of UNW. Strictly speaking, “ultrathin” is not a scientific expression, as it does not refer to a specific size range. UNWs are usually considered to be less than 10 nm in diameters in many papers, while some researchers concern the size down to sub‐5 or sub‐2 nm. Here we have no intend to argue which definition of size is more accurate, we focus on the chemical aspects of UNW. The debate of “ultrathin” may be ended one day "when … approximate practical methods of applying quantum mechanics should be developed”.^159^

Synthesis is always the first step for material realization. There are mainly three categories of fabrication methods in general: template assist, ligand control and oriented attachment synthesis.^160^ The template assist synthesis involves the use of a hard or soft structure as template, which could limit the space of nanowire growth. For example, calix(4)hydroquinone could self‐assemble to nanotubes with diameter down to atomic scale, then reducing Ag salts in the space‐limited nanotube would produce ultrathin Ag nanowire with 0.4 nm width.^161^ Soft template may occur during the synthesis, oleylamine is a kind of amine with long alkyl chains and a C‐C double bond in the middle of the chain, which in turn would allow for the easier to self‐assemble in certain condition, and Au nanowires with diameter about 1.6 nm could be generate in the channel.^162^, ^163^ Introducing the triisopropylsilane into the synthesis could accelerate the reaction with slightly changing the diameter to 1.8 nm.^164^ The principle for ligand control synthesis lies on that one ligand binds very strongly on the sides of the nanostructure and greatly inhibit the growth along this direction, thus lead to the wire formation.^165^ Oriented attachment may result in UNWs with constant diameter, the process is similar with polymerization reactions. SnO_2_ ultrathin nanowire with diameter of ca. 0.5–2.5 and ca. 1.5–4.5 nm could be prepared via the oriented attachment of SnO_2_ quantum dots.^166^ An etching method of Te nanowires could also help to fabricate several kinds of metal telluride UNWs.^167^ There is no limitation of fabrication method, more and more efficient strategies may be achieved and the rationale of fabrication may be unraveled in the future.

**Figure 10:**
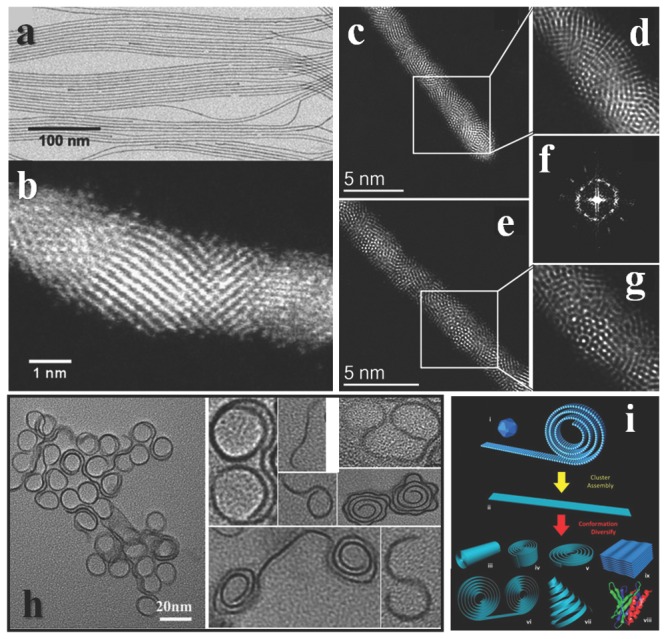
Structures of UNWs. a) TEM and b) STEM image of Au UNWs. Reproduced with permission.^174^ Copyright 2014, American Chemical Society. c,e) Aberration‐corrected STEM‐HAADF images of the Ag/Au nanowires with the helical icosahedral structure in different orientations. d,g) Corresponding high‐magnification areas of the icosahedral packing. f) FFT obtained along the axis of the nanowire of (c) showing the 5‐fold symmetry of Au nanowires. Reproduced with permission.^175^ Copyright 2011, American Chemical Society. h) The folding of ultrathin inorganic materials. Reproduced with permission.^171^ Copyright 2013, American Chemical Society. Schematic of 2D cluster‐assembly and possible variations in the ultrathin nanoribbon conformation. Reproduced with permission.^170^ Copyright 2013, American Chemical Society.

The successful synthesis put the field forward to the novel phenomena related with the ultrathin size.^168^ The relative orientations and the volume fractions of the planar defect could determine the elastic modulus of nanowire.^169^ However when the sizes of nanowires narrow down to ultrathin, it would be hard to determine what is defect. Ultrathin nanowires could be flexible, and resemble the viscosity behavior of polymers.^170^ What is more, ultrathin inorganic nanocoils may mimic the self‐folding behavior of biomacromolecues (**Figure** **10**h,i).^171^ As for metal, the structure could be changed dramatically when size down to atomic level, helicity has been obtained in Au nanowires^172^ and Pt nanotubes.^173^

It is the structure that determines the properties. However, the details of ultrathin structure remain a challenge to study. Notwithstanding, TEM is a powerful tool to characterize the morphology, and with the help of energy disperse spectrum or other accessories, element mapping or even atomic element mapping could be realized. The higher resolution usually ties to high voltage electron beam, but ultrathin structures usually cannot withstand such high energy electron beam. Such contradiction greatly hinders the structure analysis of ultrathin nanowire. Nowadays, people are making progress. High‐angle annular dark‐field (HAADF) imaging at low voltage could reduce the damage of materials under characterization. Gold nanowire with diameter about 1.7 nm was successfully analyzed under such technique. The nanowire is not atomic uniform along the wire, and the surface is not flat (Figure 10a, b).^174^ The Ag‐Au alloy UNWs possess more complete structures with icosahedral and decahedral packing along the wire (Figure 10c–g).^175^ The atomic structure of metal oxide UNWs might be more intricate, because their constitutions are even more complicated.

One possible factor that edge sites are active centers is the unsaturated coordination as we have discussed before. UNW may expose vast unsaturated coordinated atoms according to geometry, however, the vast experiments on different types of UNW are still lack, thus the researches of UNW are hindered. When size extremely narrows down, the boundary between molecule and crystal blurs. It would be hard to distinguish defects and conformation in ultrathin nanowires. Maybe we could use some knowledge from the statistics of polymer science, and build up a practical statistic explanation of the conformation in ultrathin nanowires. One more thing we need to notice is that the UNW may be not stable without capping agent. The strong binding ligands may prevent the abilities of ultrathin nanowires. Moreover, the use of UNW as catalyst is seldom reported, thus the better understanding of performance is hampered. In short, there is still a long way to realize the ultrathin nanowire as high active catalyst.

## 5 Looking Beyond Catalysts: Edges of Graphene

C is probably the most intriguing element in the periodic table. Regardless of that life is based on C, materials made by C have been used since ancient time, such as the “lead” in a pencil, fuel for fire, doping in iron. Now C materials revive once again, in names of graphene, carbon nanotube, etc. Among all the allotropes of C, graphene have become a star since the preparation by repeated peeling at 2004,^176^ because of the potential in overcoming the bottleneck of silica materials in semiconductor industry. Graphene is a honeycomb two dimensional semimetal materials with zero bandgap. The zero bandgap results in the inability of switching off for logic applications,^177^ which is deadly for application. One of the solutions is to constrain graphene nanoribbons (GNRs) with regular edges. The theoretical explanations for opening the bandgap, in short, are the linear nature of the energy dispersion near the edges of the Brillouin zone, and different boundary conditions of wave functions imposed at the edges.^178^ Thus the edge structure would determine many physical properties, like optical,^179^, ^180^ magnetic,^178^ electronic^181^, ^182^, ^183^ properties.

Here we briefly review the types of edges, synthesis methods and electrochemical properties of edges in graphene. There are several types of edge structures, including achiral and chiral edges, stable, metastable and transition structures.^184^ Though there is no perfect method to synthesis high quality graphene with perfect edge structure yet, the fabrication technique is making progress.^185^ As for researches of chemical difference between edges and basal plane, electrochemical properties are powerful evidence, though there have been so few studies.^186^

**Figure 11:**
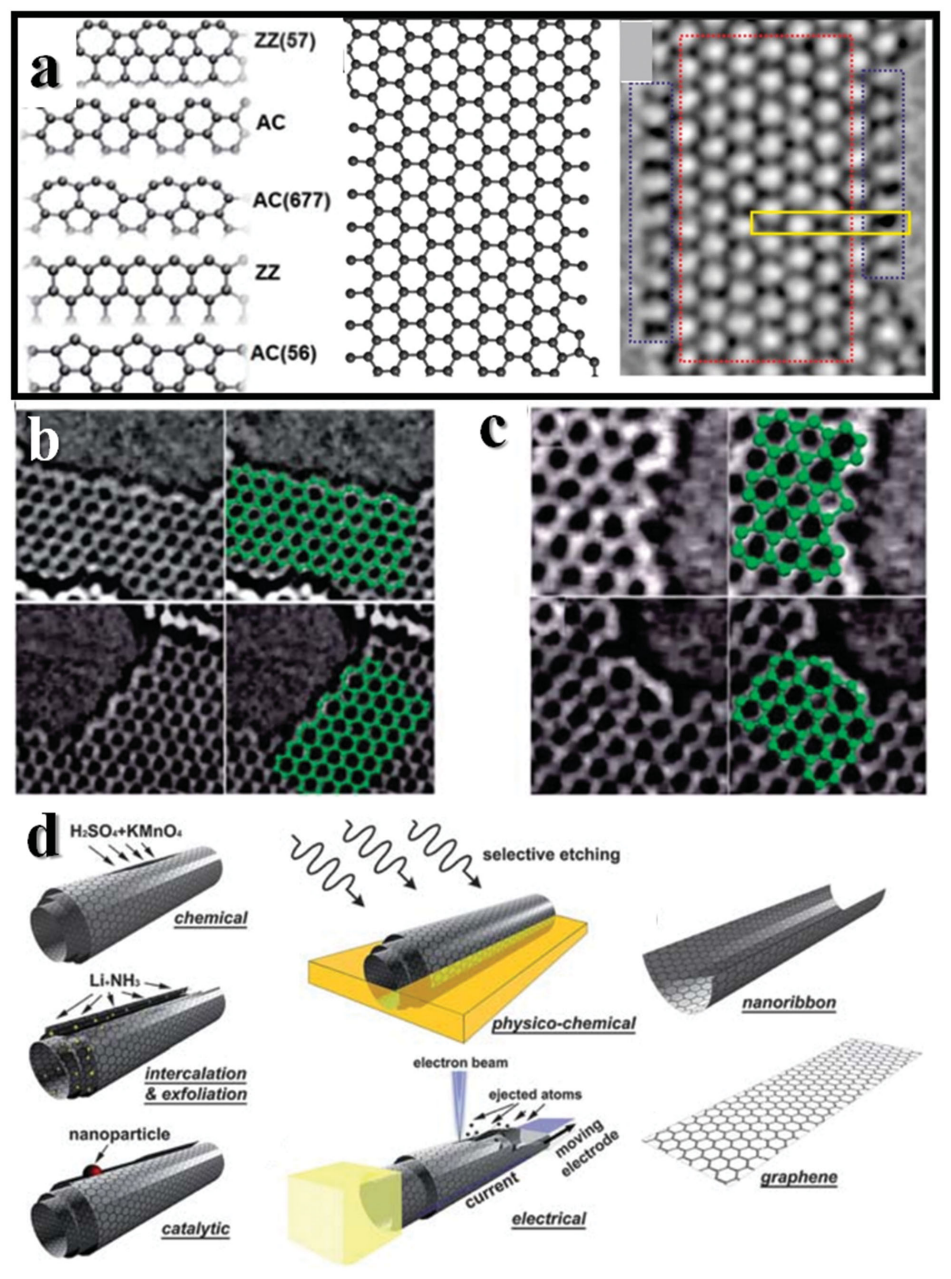
Edge structures of graphene and the fabrication of GNR. a) The geometries of graphene edges: (from top to bottom) reconstructed zigzag [ZZ(57)], armchair, reconstructed armchair [AC(677)], zigzag, and pentagonal armchair [AC(56)]. In the right is structure of EK edges. b) Experimental evidence of zigzag reconstruction. c) Experimental evidence of armchair edge reconstruction. Reproduced with premission.^185^, ^191^ Copyright 2013, Royal Society of Chemistry; Copyright 2014, American Chemical Society. d) Schematic representation of the method used for unzipping carbon nanotubes to form graphene nanoribbons. Reproduced with permission.^184^ Copyright 2011, Royal Society of Chemistry.

In general, there are two types of achiral edges in GRNs, one is zigzag GNR (ZGNR), the other is armchair GNR (AGNR), based on the orientation of hexagons (**Figure** **11**a–c). These are two most seen edge structures after Joule heating of graphene.^187^ The chiral‐GNR (CGNR) is composed by a combination of armchair and zigzag sites. The zigzag edges are metastable and easy to reconstruction into pentagon‐heptagon configurations at room temperature based on theoretical calculations.^188^ The chemical natures of zigzag sites and armchair sites were long under debating that whether there are substituent groups. However, a complete study of theoretical and experimental studies from various standpoints shown that the zigzag sites are carbene‐like and armchair sites are carbyne‐like structure with neither H‐terminated nor unadulterated free σ radicals.^189^ Another type of edges consists of series of dangling C atoms protruding from a zigzag edges, is called extended Klein (EK) edge, which is assumed unstable.^190^ EK edges have been observed in recent studies under aberration‐corrected transmission electron microscopy (AC‐TEM), results illustrate that they would turn back to zigzag edge structures or reconstruction to pentagonal edge structures in short time.^191^

One thing need to be really careful about the characterization with TEM is the reconstruction under high energy electron beams. For example, the maximum energy of an 80 keV incident electron that can be transferred to a carbon atom is 15.8 eV, and the knock‐on energy threshold for ejection of an in‐lattice carbon atom with three bonds is 17 eV. It seems safe to observe edge structure under such condition, however, the threshold drops below to 15 eV for sites with a neighboring vacancy.^192^ Such concern may expand to characterization in other nanomaterials. As for using STM and AFM to obtain edge structures, the low scan speed to get high resolution is really a risk for stability, the substrate and temperature would also influence the image of real atom arrangement. The spectrum method may be a good solution for mild and efficient characterization.^193^ All carbons show common features in their Raman spectra, the peaks lied at around 1580 and 1350 cm^−1^ are called G and D peaks respectively. For ideal zigzag edges, the D peak is zero, and large for armchair in principle. This implies the possibility to characterize the edge structure simply by Raman spectra, however, the real sample does not show such obvious feature.^194^

There are many fabrication method for GNR, including mechanical or chemical methods. Among all the methods, chemical vapor deposition (CVD) is benefit for large amount synthesis in a relative short time, and the deposited GNR can be easily transferred onto other substrate. The growth of graphene by CVD might follow the classic crystal growth theory.^195^ During CVD growth, C atoms are incorporated on the edges from the catalyst surface, however, the details at atomic scale are still needed to be explored. The shapes of graphene prepared by CVD are determined by the catalysts and other parameters, as well as the edge structure,^196^ since the graphene edge may be terminated by catalyst atoms. For example, the Cu terminated zigzag edge dramatically reduces the threshold barrier to add carbon atoms from 2.47 to 0.80 eV, thus lead to a fast growth of armchair edge.^197^

Unzipping CNTs along their longitudinal direction is an efficient method to prepare regular edge structure. Several kinds of unzipping methods are developed in various condition (Figure 11d). Multi‐wall CNTs can be unzipped in the oxidative condition with H_2_SO_4_ and KMnO_4_,^198^ or by intercalation and exfoliation with alkali‐metal and NH_3_.^199^ Cutting graphene with catalyst NPs like thermally activated nickel NPs can also produce smooth and orientated graphene edges.^200^ Other methods using electron or other beam could also be utilized to unzip the CNTs.^201^ The as prepared GNRs could be further modified using STM lithography,^202^ chemical etching.^203^ The etching strategy can extend to modify the structure of MoS_2_.^204^ Meanwhile, another “bottom‐up” method is emerging, the on‐surface polymerization from specific small molecules to GNR is a fascinating strategy to control GNR structure.^205^

Modifying the edge structure with different heteroatoms would also help to tune the bandgap.^206^ Both the covalent or noncovalent bonding at edges are available for decoration^207^, ^208^ via various methods.^209^ Readers could find a comprehensive review written by Georgakilas et al.^210^ Functionalization of graphene is of great interest in chemistry, and the well‐functionalized graphene expand their potential in various applications.^211^

The chemical environments of basal plane and edge are different, one can foresee that their electrochemical properties are not the same. For instance, metal nanowire^212^ or metal oxide nanowire^213^ can be deposited at the edges of graphite instead of on the basal plane. However, direct test of edge is of extremely difficult because of the obstacle to fabricate electrode on edges and nearly inevitable interference of basal plane. By applying CV for the oxidation of ferrocyanide and KCl solution at highly orient pyrolytic graphite (HOPG) electrode, on behalf of basal plane, and at an edge plane pyrolytic graphite electrode, the calculated electron transfer‐rate constant of edge is 7 orders of magnitude higher than the basal plane.^214^ In situ AFM^215^ and high resolution atomic force electrochemical microscopy^216^ illustrate that the basal plane is not totally inert, the electron can also transfer at pristine graphic basal plane. A recent electrochemical test using region‐specific cover with non‐conducting pinhole‐free thin film on single layer graphene sheets reveals that the edge is over 4 orders of magnitude higher specific capacitance, and has faster electron transfer rate than basal plane.^217^

## 6 Conclusion

In conclusion, edges are important parts of particles. The atoms on edge sites may be more active than atoms of facets. There are several reasons for this, including different chemical environments compared with atoms on inner surfaces, which may also lead to charge redistribution at interface, low CN. Research on MoS_2_ and related materials clearly shows that it is the edges that promote hydrogen evolution. Further studies to enhance the activity should be carried out focusing on the tuning of edge structures. Inverse catalysts represent another aspect of edge sites: the edge of an interface. The cooperation of various components around the interface can help to realize new reactions or to optimize low efficiency reactions. Edges of nanoparticles may also serve as highly active centers for reactions. Two kinds of novel structures, NFs and UNWs, are considered as atypical edges. It is not surprising that NFs have unique catalytic activities. However, the catalytic performances of UNWs are less studied yet. When the size of UNWs approach the molecular level, the boundary of the crystal and molecule should be reconsidered. Looking beyond the edges in catalysts, the edge structure of graphene is also essential for their physical or chemical properties. The band structures of zigzag or armchair edge sites in graphene are quite different, and this is also true for other 2D materials, such as MoS_2_.^218^ In short, the edges of two dimensional materials are pivotal for their applications.

Despite the great success in material fabrication, there are still many obstacles that hinder the understanding of nano­world. As size decreases, the characterization of materials become difficult, mainly because of the inherent instability of materials. Our limited understanding of crystals or solids at sizes near the molecular level may also hamper the realization of applications. Furthermore, the identification of the active center in heterogeneous catalyst is still a challenge in many cases due to the lack of proper method to track the reactions. Although computer‐aided calculations have achieved great success in detailing many reactions, they usually require a long time to achieve suitable accuracy. Therefore further research, both experimental and theoretical, needs to be done to decipher the structures and properties of nanomaterials in the future.

## References

[1] H. Tong , S. Ouyang , Y. Bi , N. Umezawa , M. Oshikiri , J. Ye , Adv. Mater. 2012, 24, 229. 21972044 10.1002/adma.201102752

[2] R. Narayanan , M. A. El‐Sayed , J. Am. Chem. Soc. 2003, 125, 8340. 12837106 10.1021/ja035044x

[3] P. V. Kamat , Chem. Rev. 1993, 93, 267.

[4] W. Zhu , R. Michalsky , Ö. Metin , H. Lv , S. Guo , C. J. Wright , X. Sun , A. A. Peterson , S. Sun , J. Am. Chem. Soc. 2013, 135, 16833. 24156631 10.1021/ja409445p

[5] C.‐H. Cui , S.‐H. Yu , Acc. Chem. Res. 2013, 46, 1427. 23425040 10.1021/ar300254b

[6] C. Burda , X. Chen , R. Narayanan , M. A. El‐Sayed , Chem. Rev. 2005, 105, 1025. 15826010 10.1021/cr030063a

[7] J. C. Love , L. A. Estroff , J. K. Kriebel , R. G. Nuzzo , G. M. Whitesides , Chem. Rev. 2005, 105, 1103. 15826011 10.1021/cr0300789

[8] S. T. Ceyer , Annu. Rev. Phys. Chem. 1988, 39, 479.

[9] P. Raybaud , J. Hafner , G. Kresse , S. Kasztelan , H. Toulhoat , J. Catal. 2000, 189, 129.

[10] A. H. Castro Neto , F. Guinea , N. M. R. Peres , K. S. Novoselov , A. K. Geim , Rev. Mod. Phys. 2009, 81, 109.

[11] N. T. S. Phan , M. Van Der Sluys , C. W. Jones , Adv. Synth. Catal. 2006, 348, 609.

[12] V. Polshettiwar , R. S. Varma , Green Chem. 2010, 12, 743.

[13] S. B. Kalidindi , B. R. Jagirdar , ChemSusChem 2012, 5, 65. 22190344 10.1002/cssc.201100377

[14] D. Astruc , F. Lu , J. R. Aranzaes , Angew. Chem. Int. Ed. 2005, 44, 7852. 10.1002/anie.200500766 16304662

[15] Y. Li , Sci. China Chem. 2014, 924.

[16] X. Guo , G. Fang , G. Li , H. Ma , H. Fan , L. Yu , C. Ma , X. Wu , D. Deng , M. Wei , D. Tan , R. Si , S. Zhang , J. Li , L. Sun , Z. Tang , X. Pan , X. Bao , Science 2014, 344, 616. 24812398 10.1126/science.1253150

[17] A. T. Bell , Sci. China Chem. 2014, 923.

[18] M. A. Lukowski , A. S. Daniel , F. Meng , A. Forticaux , L. Li , S. Jin , J. Am. Chem. Soc. 2013, 135, 10274. 23790049 10.1021/ja404523s

[19] Y. Hao , S. Ji , X. Liu , D. Zhang , D. Shi , Sci. China Chem. 2014, 57, 866.

[20] Y. Ji , L. Wu , Q. Fan , Acta Chim. Sinica 2014, 72, 798.

[21] R. Schlögl , S. B. Abd Hamid , Angew. Chem. Int. Ed. 2004, 43, 1628. 10.1002/anie.200301684 15038028

[22] Y.‐G. Wang , Y. Yoon , V.‐A. Glezakou , J. Li , R. Rousseau , J. Am. Chem. Soc. 2013, 135, 10673. 23782230 10.1021/ja402063v

[23] Y. Lu , Y. Jiang , X. Gao , X. Wang , W. Chen , J. Am. Chem. Soc. 2014, 136, 11687. 25054583 10.1021/ja5041094

[24] H. Zeuthen , W. Kudernatsch , L. R. Merte , L. K. Ono , L. Lammich , F. Besenbacher , S. Wendt , ACS Nano 2015, 9, 573. 25574971 10.1021/nn505890v

[25] Z. Xi , Acc. Chem. Res. 2010, 43, 1342. 20954749 10.1021/ar1000583

[26] B. K. Burgess , D. J. Lowe , Chem. Rev. 1996, 96, 2983. 11848849 10.1021/cr950055x

[27] K. Semba , Y. Nakao , J. Am. Chem. Soc. 2014, 136, 7567. 24810227 10.1021/ja5029556

[28] B. Xiao , Z. Niu , Y.‐G. Wang , W. Jia , J. Shang , L. Zhang , D. Wang , Y. Fu , J. Zeng , W. He , K. Wu , J. Li , J. Yang , L. Liu , Y. Li , J. Am. Chem. Soc. 2015, 137, 3791. 25778784 10.1021/jacs.5b01391

[29] Z.‐P. Liu , P. Hu , J. Am. Chem. Soc. 2003, 125, 1958.12580623

[30] Y. Yan , B. Xia , Z. Xu , X. Wang , ACS Catal. 2014, 4, 1693.

[31] M. G. Walter , E. L. Warren , J. R. McKone , S. W. Boettcher , Q. Mi , E. A. Santori , N. S. Lewis , Chem. Rev. 2010, 110, 6446. 21062097 10.1021/cr1002326

[32] J. Greeley , T. F. Jaramillo , J. Bonde , I. Chorkendorff , J. K. Norskov , Nat. Mater. 2006, 5, 909. 17041585 10.1038/nmat1752

[33] D. C. Rees , J. B. Howard , Science 2003, 300, 929. 12738849 10.1126/science.1083075

[34] D. J. Evans , C. J. Pickett , Chem. Soc. Rev. 2003, 32, 268. 14518180 10.1039/b201317g

[35] B. Hinnemann , P. G. Moses , J. Bonde , K. P. Jørgensen , J. H. Nielsen , S. Horch , I. Chorkendorff , J. K. Nørskov , J. Am. Chem. Soc. 2005, 127, 5308. 15826154 10.1021/ja0504690

[36] T. F. Jaramillo , K. P. Jørgensen , J. Bonde , J. H. Nielsen , S. Horch , I. Chorkendorff , Science 2007, 317, 100. 17615351 10.1126/science.1141483

[37] S. Helveg , J. V. Lauritsen , E. Lægsgaard , I. Stensgaard , J. K. Nørskov , B. S. Clausen , H. Topsøe , F. Besenbacher , Phys. Rev. Lett. 2000, 84, 951. 11017413 10.1103/PhysRevLett.84.951

[38] M. Xu , T. Liang , M. Shi , H. Chen , Chem. Rev. 2013, 113, 3766. 23286380 10.1021/cr300263a

[39] L. P. Hansen , Q. M. Ramasse , C. Kisielowski , M. Brorson , E. Johnson , H. Topsøe , S. Helveg , Angew. Chem. Int. Ed. 2011, 50, 10153. 10.1002/anie.201103745 22021210

[40] M. V. Bollinger , J. V. Lauritsen , K. W. Jacobsen , J. K. Nørskov , S. Helveg , F. Besenbacher , Phys. Rev. Lett. 2001, 87, 196803. 11690441 10.1103/PhysRevLett.87.196803

[41] L. Byskov , J. Nørskov , B. Clausen , H. Topsøe , Catal. Lett. 2000, 64, 95.

[42] J. V. Lauritsen , J. Kibsgaard , S. Helveg , H. Topsoe , B. S. Clausen , E. Laegsgaard , F. Besenbacher , Nat Nanotechnol. 2007, 2, 53. 18654208 10.1038/nnano.2006.171

[43] M. Chhowalla , H. S. Shin , G. Eda , L.‐J. Li , K. P. Loh , H. Zhang , Nat. Chem. 2013, 5, 263. 23511414 10.1038/nchem.1589

[44] A. Albu‐Yaron , M. Levy , R. Tenne , R. Popovitz‐Biro , M. Weidenbach , M. Bar‐Sadan , L. Houben , A. N. Enyashin , G. Seifert , D. Feuermann , E. A. Katz , J. M. Gordon , Angew. Chem. Int. Ed. 2011, 50, 1810. 10.1002/anie.201006719 21328645

[45] A. B. Laursen , S. Kegnaes , S. Dahl , I. Chorkendorff , Energy Environ. Sci. 2012, 5, 5577.

[46] H. I. Karunadasa , E. Montalvo , Y. Sun , M. Majda , J. R. Long , C. J. Chang , Science 2012, 335, 698. 22323816 10.1126/science.1215868

[47] J. Kibsgaard , Z. Chen , B. N. Reinecke , T. F. Jaramillo , Nat. Mater. 2012, 11, 963. 23042413 10.1038/nmat3439

[48] J. Xie , H. Zhang , S. Li , R. Wang , X. Sun , M. Zhou , J. Zhou , X. W. Lou , Y. Xie , Adv. Mater. 2013, 25, 5807. 23943511 10.1002/adma.201302685

[49] Y. Li , H. Wang , L. Xie , Y. Liang , G. Hong , H. Dai , J. Am. Chem. Soc. 2011, 133, 7296. 21510646 10.1021/ja201269b

[50] V. Senthilkumar , L. Tam , Y. Kim , Y. Sim , M.‐J. Seong , J. I. Jang , Nano Res. 2014, 7, 1759.

[51] D. Kong , H. Wang , J. J. Cha , M. Pasta , K. J. Koski , J. Yao , Y. Cui , Nano Lett. 2013, 13, 1341. 23387444 10.1021/nl400258t

[52] H. Wang , Q. Zhang , H. Yao , Z. Liang , H.‐W. Lee , P.‐C. Hsu , G. Zheng , Y. Cui , Nano Lett. 2014, 14, 7138. 25372985 10.1021/nl503730c

[53] H. Wang , C. Tsai , D. Kong , K. Chan , F. Abild‐Pedersen , J. Nørskov , Y. Cui , Nano Res. 2015, 8, 566.

[54] M.‐R. Gao , J.‐X. Liang , Y.‐R. Zheng , Y.‐F. Xu , J. Jiang , Q. Gao , J. Li , S.‐H. Yu , Nat. Commun. 2015, 6, 5982. 25585911 10.1038/ncomms6982 PMC4309426

[55] X. Wang , B. Ni , Chem. Sci. 2015, doi: 10.1039/C5SC00836K.

[56] F. Meng , M. S. A. F. Audrey , J. Song , Acc. Chem. Res. 2013, 46, 1616. 23738750 10.1021/ar400003q

[57] L. Chen , B. Liu , A. N. Abbas , Y. Ma , X. Fang , Y. Liu , C. Zhou , ACS Nano 2014, 8, 11543. 25350314 10.1021/nn504775f

[58] L. Zhang , K. Liu , A. B. Wong , J. Kim , X. Hong , C. Liu , T. Cao , S. G. Louie , F. Wang , P. Yang , Nano Lett. 2014, 14, 6418. 25343743 10.1021/nl502961e

[59] Q. Zhu , S. L. Wegener , C. Xie , O. Uche , M. Neurock , T. J. Marks , Nat. Chem. 2013, 5, 104. 23344430 10.1038/nchem.1527

[60] D. Voiry , H. Yamaguchi , J. Li , R. Silva , D. C. B. Alves , T. Fujita , M. Chen , T. Asefa , V. B. Shenoy , G. Eda , M. Chhowalla , Nat. Mater. 2013, 12, 850. 23832127 10.1038/nmat3700

[61] C. Choi , J. Feng , Y. Li , J. Wu , A. Zak , R. Tenne , H. Dai , Nano Res. 2013, 6, 921.

[62] C. Tsai , K. Chan , F. Abild‐Pedersen , J. K. Norskov , PCCP 2014, 16, 13156. 24866567 10.1039/c4cp01237b

[63] A. S. K. Hashmi , G. J. Hutchings , Angew. Chem. Int. Ed. 2006, 45, 7896. 10.1002/anie.200602454 17131371

[64] M. Haruta , T. Kobayashi , H. Sano , N. Yamada , Chem. Lett. 1987, 16, 405.

[65] A. Goeppert , M. Czaun , R. B. May , G. K. S. Prakash , G. A. Olah , S. R. Narayanan , J. Am. Chem. Soc. 2011, 133, 20164. 22103291 10.1021/ja2100005

[66] J. Bonin , M. Robert , M. Routier , J. Am. Chem. Soc. 2014, 136, 16768. 25396278 10.1021/ja510290t

[67] R. K. Yadav , G. H. Oh , N.‐J. Park , A. Kumar , K.‐j. Kong , J.‐O. Baeg , J. Am. Chem. Soc. 2014, 136, 16728. 25405924 10.1021/ja509650r

[68] X. Li , J. Wen , J. Low , Y. Fang , J. Yu , Sci. China Mater. 2014, 57, 70.

[69] A. A. Peterson , J. K. Nørskov , J. Phys. Chem. Lett. 2012, 3, 251.

[70] A. A. Peterson , F. Abild‐Pedersen , F. Studt , J. Rossmeisl , J. K. Norskov , Energy Environ. Sci. 2010, 3, 1311.

[71] W. J. Durand , A. A. Peterson , F. Studt , F. Abild‐Pedersen , J. K. Nørskov , Surf. Sci. 2011, 605, 1354.

[72] H. A. Hansen , J. B. Varley , A. A. Peterson , J. K. Nørskov , J. Phys. Chem. Lett. 2013, 4, 388. 26281729 10.1021/jz3021155

[73] D. R. Kauffman , D. Alfonso , C. Matranga , H. Qian , R. Jin , J. Am. Chem. Soc. 2012, 134, 10237. 22616945 10.1021/ja303259q

[74] H. Mistry , R. Reske , Z. Zeng , Z.‐J. Zhao , J. Greeley , P. Strasser , B. R. Cuenya , J. Am. Chem. Soc. 2014, 136, 16473. 25325519 10.1021/ja508879j

[75] W. Zhu , Y.‐J. Zhang , H. Zhang , H. Lv , Q. Li , R. Michalsky , A. A. Peterson , S. Sun , J. Am. Chem. Soc. 2014, 136, 16132. 25380393 10.1021/ja5095099

[76] G. Ertl , M. Neumann , K. M. Streit , Surf. Sci. 1977, 64, 393.

[77] Y. Y. Yeo , L. Vattuone , D. A. King , J. Chem. Phys. 1997, 106, 392.

[78] J.‐H. Fischer‐Wolfarth , J. Hartmann , J. A. Farmer , J. M. Flores‐Camacho , C. T. Campbell , S. Schauermann , H.‐J. Freund , Rev. Sci. Instrum. 2011, 82, 024102. 21361615 10.1063/1.3544020

[79] K. Golibrzuch , P. R. Shirhatti , J. Geweke , J. Werdecker , A. Kandratsenka , D. J. Auerbach , A. M. Wodtke , C. Bartels , J. Am. Chem. Soc. 2014, 137, 1465. 10.1021/ja509530k 25436871

[80] K. P. Kuhl , T. Hatsukade , E. R. Cave , D. N. Abram , J. Kibsgaard , T. F. Jaramillo , J. Am. Chem. Soc. 2014, 136, 14107. 25259478 10.1021/ja505791r

[81] K. J. P. Schouten , Y. Kwon , C. J. van der Ham , Z. Qin , M. T. M. Koper , Chem. Sci. 2011, 2, 1902.

[82] K. J. P. Schouten , Z. Qin , E. P. Gallent , M. T. M. Koper , J. Am. Chem. Soc. 2012, 134, 9864. 22670713 10.1021/ja302668n

[83] Y.‐G. Kim , J. H. Baricuatro , A. Javier , J. M. Gregoire , M. P. Soriaga , Langmuir 2014, 30, 15053. 25489793 10.1021/la504445g

[84] H.‐K. Lim , H. Shin , W. A. Goddard , Y. J. Hwang , B. K. Min , H. Kim , J. Am. Chem. Soc. 2014, 136, 11355. 25061918 10.1021/ja503782w

[85] K. Manthiram , B. J. Beberwyck , A. P. Alivisatos , J. Am. Chem. Soc. 2014, 136, 13319. 25137433 10.1021/ja5065284

[86] D. Kim , J. Resasco , Y. Yu , A. M. Asiri , P. Yang , Nat. Commun. 2014, 5, 4948. 25208828 10.1038/ncomms5948

[87] S. J. Tauster , S. C. Fung , R. L. Garten , J. Am. Chem. Soc. 1978, 100, 170.

[88] S. J. Tauster , Acc. Chem. Res. 1987, 20, 389.

[89] S. Bonanni , K. Aït‐Mansour , H. Brune , W. Harbich , ACS Catal. 2011, 1, 385.

[90] L. Li , X. C. Zeng , J. Am. Chem. Soc. 2014, 136, 15857. 25338299 10.1021/ja508666a

[91] B. Y. Xia , H. B. Wu , Y. Yan , H. B. Wang , X. Wang , Small 2014, 10, 2336. 24610604 10.1002/smll.201302648

[92] R. Subbaraman , D. Tripkovic , D. Strmcnik , K.‐C. Chang , M. Uchimura , A. P. Paulikas , V. Stamenkovic , N. M. Markovic , Science 2011, 334, 1256. 22144621 10.1126/science.1211934

[93] Q. Fu , F. Yang , X. Bao , Acc. Chem. Res. 2013, 46, 1692. 23458033 10.1021/ar300249b

[94] Q. Fu , W.‐X. Li , Y. Yao , H. Liu , H.‐Y. Su , D. Ma , X.‐K. Gu , L. Chen , Z. Wang , H. Zhang , B. Wang , X. Bao , Science 2010, 328, 1141. 20508127 10.1126/science.1188267

[95] R. Mu , X. Guo , Q. Fu , X. Bao , J. Phys. Chem. C 2011, 115, 20590.

[96] R. Mu , Q. Fu , H. Xu , H. Zhang , Y. Huang , Z. Jiang , S. Zhang , D. Tan , X. Bao , J. Am. Chem. Soc. 2011, 133, 1978. 21247156 10.1021/ja109483a

[97] H. Xu , Q. Fu , X. Guo , X. Bao , ChemCatChem 2012, 4, 1645.

[98] Y. Yao , Q. Fu , Z. Wang , D. Tan , X. Bao , J. Phys. Chem. C 2010, 114, 17069.

[99] Y. Wang , H. Zhang , X. Yao , H. Zhao , J. Phys. Chem. C 2013, 117, 1672.

[100] X.‐K. Gu , R. Ouyang , D. Sun , H.‐Y. Su , W.‐X. Li , ChemSusChem 2012, 5, 871. 22162485 10.1002/cssc.201100525

[101] W. Wang , H. Zhang , W. Wang , A. Zhao , B. Wang , J. G. Hou , Chem. Phys. Lett. 2010, 500, 76.

[102] L. R. Merte , J. Knudsen , L. C. Grabow , R. T. Vang , E. Lægsgaard , M. Mavrikakis , F. Besenbacher , Surf. Sci. 2009, 603, L15.

[103] W. Huang , W. Ranke , Surf. Sci. 2006, 600, 793.

[104] L. Xu , Z. Wu , Y. Zhang , B. Chen , Z. Jiang , Y. Ma , W. Huang , J. Phys. Chem. C 2011, 115, 14290.

[105] J. A. Rodriguez , S. Ma , P. Liu , J. Hrbek , J. Evans , M. Pérez , Science 2007, 318, 1757. 18079397 10.1126/science.1150038

[106] G. Ketteler , W. Ranke , J. Phys. Chem. B 2003, 107, 4320.

[107] C. Ebensperger , M. Gubo , W. Meyer , L. Hammer , K. Heinz , Phys. Rev. B 2010, 81, 235405.

[108] T. Franz , J. Zabloudil , F. Mittendorfer , L. Gragnaniello , G. Parteder , F. Allegretti , S. Surnev , F. P. Netzer , J. Phys. Chem. Lett. 2011, 3, 186.

[109] H.‐J. Freund , G. Pacchioni , Chem. Soc. Rev. 2008, 37, 2224. 18818825 10.1039/b718768h

[110] S. Surnev , A. Fortunelli , F. P. Netzer , Chem. Rev. 2012, 113, 4314. 23237602 10.1021/cr300307n

[111] K. An , S. Alayoglu , N. Musselwhite , S. Plamthottam , G. Melaet , A. E. Lindeman , G. A. Somorjai , J. Am. Chem. Soc. 2013, 135, 16689. 24090187 10.1021/ja4088743

[112] M. G. Willinger , W. Zhang , O. Bondarchuk , S. Shaikhutdinov , H.‐J. Freund , R. Schlögl , Angew. Chem. Int. Ed. 2014, 53, 5998. 10.1002/anie.201400290 24840397

[113] P. Liu , X. Ge , R. Wang , H. Ma , Y. Ding , Langmuir 2009, 25, 561. 19063640 10.1021/la8027034

[114] Y. Bai , W. Zhang , Z. Zhang , J. Zhou , X. Wang , C. Wang , W. Huang , J. Jiang , Y. Xiong , J. Am. Chem. Soc. 2014, 136, 14650. 25296380 10.1021/ja506269y

[115] D. Matthey , J. G. Wang , S. Wendt , J. Matthiesen , R. Schaub , E. Lægsgaard , B. Hammer , F. Besenbacher , Science 2007, 315, 1692. 17379802 10.1126/science.1135752

[116] I. X. Green , W. Tang , M. Neurock , J. T. Yates , Science 2011, 333, 736. 21817048 10.1126/science.1207272

[117] M. McEntee , W. Tang , M. Neurock , J. T. Yates , J. Am. Chem. Soc. 2014, 136, 5116. 24597473 10.1021/ja500928h

[118] B. Y. Xia , H. B. Wu , X. Wang , X. W. Lou , Angew. Chem. 2013, 125, 12563.

[119] B. Y. Xia , H. B. Wu , Y. Yan , X. W. Lou , X. Wang , J. Am. Chem. Soc. 2013, 135, 9480. 23742152 10.1021/ja402955t

[120] B. Y. Xia , H. B. Wu , N. Li , Y. Yan , X. W. Lou , X. Wang , Angew. Chem. 2015, 127, 3868. 10.1002/anie.201411544 25630856

[121] H. Zhang , M. Jin , Y. Xia , Angew. Chem. Int. Ed. 2012, 51, 7656. 10.1002/anie.201201557 22639064

[122] H. Zhang , W. Li , M. Jin , J. Zeng , T. Yu , D. Yang , Y. Xia , Nano Lett. 2010, 11, 898. 21192673 10.1021/nl104347j

[123] M. Jin , H. Zhang , Z. Xie , Y. Xia , Angew. Chem. Int. Ed. 2011, 50, 7850. 10.1002/anie.201103002 21732512

[124] X. Xia , J. Zeng , B. McDearmon , Y. Zheng , Q. Li , Y. Xia , Angew. Chem. Int. Ed. 2011, 50, 12542. 10.1002/anie.201105200 21913296

[125] J. Zhang , M. R. Langille , M. L. Personick , K. Zhang , S. Li , C. A. Mirkin , J. Am. Chem. Soc. 2010, 132, 14012. 20853848 10.1021/ja106394k

[126] X. Huang , Z. Zhao , J. Fan , Y. Tan , N. Zheng , J. Am. Chem. Soc. 2011, 133, 4718. 21405136 10.1021/ja1117528

[127] M. V. Kovalenko , L. Manna , A. Cabot , Z. Hens , D. V. Talapin , C. R. Kagan , V. I. Klimov , A. L. Rogach , P. Reiss , D. J. Milliron , P. Guyot‐Sionnnest , G. Konstantatos , W. J. Parak , T. Hyeon , B. A. Korgel , C. B. Murray , W. Heiss , ACS Nano 2015 9, 1012. 25608730 10.1021/nn506223h

[128] N. Fan , Y. Yang , W. Wang , L. Zhang , W. Chen , C. Zou , S. Huang , ACS Nano 2012, 6, 4072. 22506898 10.1021/nn3004668

[129] X. Wu , Y. Yu , Y. Liu , Y. Xu , C. Liu , B. Zhang , Angew. Chem. Int. Ed. 2012, 51, 3211. 10.1002/anie.201108098 22334529

[130] S. Xie , N. Lu , Z. Xie , J. Wang , M. J. Kim , Y. Xia , Angew. Chem. Int. Ed. 2012, 51, 10266. 10.1002/anie.201206044 22968993

[131] Z. L. Wang , J. Phys. Chem. B 2000, 104, 1153.

[132] G. S. Métraux , Y. C. Cao , R. Jin , C. A. Mirkin , Nano Lett. 2003, 3, 519.

[133] M. M. Shahjamali , M. Bosman , S. Cao , X. Huang , X. Cao , H. Zhang , S. S. Pramana , C. Xue , Small 2013, 9, 2880. 23447112 10.1002/smll.201300200

[134] M. McEachran , D. Keogh , B. Pietrobon , N. Cathcart , I. Gourevich , N. Coombs , V. Kitaev , J. Am. Chem. Soc. 2011, 133, 8066. 21557604 10.1021/ja111642d

[135] J. E. Macdonald , M. Bar Sadan , L. Houben , I. Popov , U. Banin , Nat. Mater. 2010, 9, 810. 20852616 10.1038/nmat2848

[136] C. Han , L. Ge , C. Chen , Y. Li , Z. Zhao , X. Xiao , Z. Li , J. Zhang , J. Mater. Chem. A 2014, 2, 12594.

[137] Y. Yin , R. M. Rioux , C. K. Erdonmez , S. Hughes , G. A. Somorjai , A. P. Alivisatos , Science 2004, 304, 711. 15118156 10.1126/science.1096566

[138] C. Chen , Y. Kang , Z. Huo , Z. Zhu , W. Huang , H. L. Xin , J. D. Snyder , D. Li , J. A. Herron , M. Mavrikakis , M. Chi , K. L. More , Y. Li , N. M. Markovic , G. A. Somorjai , P. Yang , V. R. Stamenkovic , Science 2014, 343, 1339. 24578531 10.1126/science.1249061

[139] Y. Wang , Y. Chen , C. Nan , L. Li , D. Wang , Q. Peng , Y. Li , Nano Res. 2015, 8, 140.

[140] Y. Wu , D. Wang , G. Zhou , R. Yu , C. Chen , Y. Li , J. Am. Chem. Soc. 2014, 136, 11594. 25090920 10.1021/ja5058532

[141] B. Y. Xia , H. B. Wu , X. Wang , X. W. Lou , J. Am. Chem. Soc. 2012, 134, 13934. 22897642 10.1021/ja3051662

[142] F. Nosheen , Z.‐c. Zhang , J. Zhuang , X. Wang , Nanoscale 2013, 5, 3660. 23552299 10.1039/c3nr00833a

[143] X. Hong , D. Wang , S. Cai , H. Rong , Y. Li , J. Am. Chem. Soc. 2012, 134, 18165. 23088493 10.1021/ja3076132

[144] X. Lu , L. Au , J. McLellan , Z.‐Y. Li , M. Marquez , Y. Xia , Nano Lett. 2007, 7, 1764. 17489641 10.1021/nl070838l PMC2504472

[145] Q. Zhang , C. M. Cobley , J. Zeng , L.‐P. Wen , J. Chen , Y. Xia , J. Phys. Chem. C 2010, 114, 6396. 10.1021/jp100354z PMC2873216 20495675

[146] S. E. Skrabalak , J. Chen , Y. Sun , X. Lu , L. Au , C. M. Cobley , Y. Xia , Acc. Chem. Res. 2008, 41, 1587. 18570442 10.1021/ar800018v PMC2645935

[147] C.‐H. Kuo , M. H. Huang , J. Am. Chem. Soc. 2008, 130, 12815. 18761449 10.1021/ja804625s

[148] Y.‐H. Tsai , C.‐Y. Chiu , M. H. Huang , J. Phys. Chem. C 2013, 117, 24611.

[149] Y. Sui , W. Fu , Y. Zeng , H. Yang , Y. Zhang , H. Chen , Y. Li , M. Li , G. Zou , Angew. Chem. Int. Ed. 2010, 49, 4282. 10.1002/anie.200907117 20446323

[150] L.‐M. Lyu , M. H. Huang , J. Phys. Chem. C 2011, 115, 17768.

[151] M. S. Yavuz , Y. Cheng , J. Chen , C. M. Cobley , Q. Zhang , M. Rycenga , J. Xie , C. Kim , K. H. Song , A. G. Schwartz , L. V. Wang , Y. Xia , Nat. Mater. 2009, 8, 935. 19881498 10.1038/nmat2564 PMC2787748

[152] M. J. Mulvihill , X. Y. Ling , J. Henzie , P. Yang , J. Am. Chem. Soc. 2009, 132, 268. 10.1021/ja906954f 20000421

[153] M. A. Mahmoud , M. A. El‐Sayed , J. Phys. Chem. C 2008, 112, 14618.

[154] M. A. Mahmoud , M. A. El‐Sayed , Nano Lett. 2009, 9, 3025. 19585987 10.1021/nl901501x

[155] M. Oezaslan , M. Heggen , P. Strasser , J. Am. Chem. Soc. 2011, 134, 514. 22129031 10.1021/ja2088162

[156] L. Su , W. Jia , C.‐M. Li , Y. Lei , ChemSusChem 2014, 7, 361. 24449484 10.1002/cssc.201300823

[157] J. Zeng , Q. Zhang , J. Chen , Y. Xia , Nano Lett. 2009, 10, 30. 10.1021/nl903062e 19928909

[158] M. A. Mahmoud , W. Qian , M. A. El‐Sayed , Nano Lett. 2011, 11, 3285. 21721552 10.1021/nl201642r

[159] P. A. M. Dirac , Proc. R. Soc. London 1929, 123, 714.

[160] L. Cademartiri , G. A. Ozin , Adv. Mater. 2009, 21, 1013.

[161] B. H. Hong , S. C. Bae , C.‐W. Lee , S. Jeong , K. S. Kim , Science 2001, 294, 348. 11546837 10.1126/science.1062126

[162] Z. Huo , C.‐k. Tsung , W. Huang , X. Zhang , P. Yang , Nano Lett. 2008, 8, 2041. 18537294 10.1021/nl8013549

[163] N. Pazos‐Pérez , D. Baranov , S. Irsen , M. Hilgendorff , L. M. Liz‐Marzán , M. Giersig , Langmuir 2008, 24, 9855. 18652498 10.1021/la801675d

[164] H. Feng , Y. Yang , Y. You , G. Li , J. Guo , T. Yu , Z. Shen , T. Wu , B. Xing , Chem. Commun. 2009, 1984. 10.1039/b822507a 19333465

[165] X. Peng , Adv. Mater. 2003, 15, 459.

[166] X. Xu , J. Zhuang , X. Wang , J. Am. Chem. Soc. 2008, 130, 12527. 18715007 10.1021/ja8040527

[167] H. Yang , S. W. Finefrock , J. D. Albarracin Caballero , Y. Wu , J. Am. Chem. Soc. 2014, 136, 10242. 25003347 10.1021/ja505304v

[168] S. Hu , X. Wang , Chem. Soc. Rev. 2013, 42, 5577. 23589105 10.1039/c3cs00006k

[169] S. Dai , J. Zhao , M.‐r. He , X. Wang , J. Wan , Z. Shan , J. Zhu , Nano Lett. 2014, 15, 8. 25427143 10.1021/nl501986d

[170] S. Hu , H. Liu , P. Wang , X. Wang , J. Am. Chem. Soc. 2013, 135, 11115. 23837618 10.1021/ja403471d

[171] P.‐p. Wang , Y. Yang , J. Zhuang , X. Wang , J. Am. Chem. Soc. 2013, 135, 6834. 23611283 10.1021/ja403065z

[172] Y. Kondo , K. Takayanagi , Science 2000, 289, 606. 10915620 10.1126/science.289.5479.606

[173] Y. Oshima , H. Koizumi , K. Mouri , H. Hirayama , K. Takayanagi , Y. Kondo , Phys. Rev. B 2002, 65, 121401.

[174] L.‐M. Lacroix , R. Arenal , G. Viau , J. Am. Chem. Soc. 2014, 136, 13075. 25188861 10.1021/ja507728j

[175] J. J. Velázquez‐Salazar , R. Esparza , S. J. Mejía‐Rosales , R. Estrada‐Salas , A. Ponce , F. L. Deepak , C. Castro‐Guerrero , M. José‐Yacamán , ACS Nano 2011, 5, 6272. 21790155 10.1021/nn202495r PMC3180901

[176] K. S. Novoselov , A. K. Geim , S. V. Morozov , D. Jiang , Y. Zhang , S. V. Dubonos , I. V. Grigorieva , A. A. Firsov , Science 2004, 306, 666. 15499015 10.1126/science.1102896

[177] F. Schwierz , Nat. Nanotechnol. 2010, 5, 487. 20512128 10.1038/nnano.2010.89

[178] D. S. L. Abergel , V. Apalkov , J. Berashevich , K. Ziegler , T. Chakraborty , Adv. Phys. 2010, 59, 261.

[179] S. Malola , H. Häkkinen , P. Koskinen , Eur. Phys. J. D 2009, 52, 71.

[180] A. V. Savin , Y. S. Kivshar , Phys. Rev. B 2010, 81, 165418.

[181] M. Y. Han , B. Özyilmaz , Y. Zhang , P. Kim , Phys. Rev. Lett. 2007, 98, 206805. 17677729 10.1103/PhysRevLett.98.206805

[182] S. M. M. Dubois , A. Lopez‐Bezanilla , A. Cresti , F. Triozon , B. Biel , J.‐C. Charlier , S. Roche , ACS Nano 2010, 4, 1971. 20355732 10.1021/nn100028q

[183] D. L. Nika , E. P. Pokatilov , A. S. Askerov , A. A. Balandin , Phys. Rev. B 2009, 79, 155413.

[184] X. Jia , J. Campos‐Delgado , M. Terrones , V. Meunier , M. S. Dresselhaus , Nanoscale 2011, 3, 86. 21103548 10.1039/c0nr00600a

[185] X. Zhang , J. Xin , F. Ding , Nanoscale 2013, 5, 2556. 23420074 10.1039/c3nr34009k

[186] W. Yang , K. R. Ratinac , S. P. Ringer , P. Thordarson , J. J. Gooding , F. Braet , Angew. Chem. Int. Ed. 2010, 49, 2114. 10.1002/anie.200903463 20187048

[187] X. Jia , M. Hofmann , V. Meunier , B. G. Sumpter , J. Campos‐Delgado , J. M. Romo‐Herrera , H. Son , Y.‐P. Hsieh , A. Reina , J. Kong , M. Terrones , M. S. Dresselhaus , Science 2009, 323, 1701. 19325109 10.1126/science.1166862

[188] P. Koskinen , S. Malola , H. Häkkinen , Phys. Rev. Lett. 2008, 101, 115502. 18851294 10.1103/PhysRevLett.101.115502

[189] L. R. Radovic , B. Bockrath , J. Am. Chem. Soc. 2005, 127, 5917. 15839691 10.1021/ja050124h

[190] D. J. Klein , L. Bytautas , J. Phys. Chem. A 1999, 103, 5196.

[191] K. He , A. W. Robertson , S. Lee , E. Yoon , G.‐D. Lee , J. H. Warner , ACS Nano 2014, 8, 12272. 25533172 10.1021/nn504471m

[192] Ç. Ö. Girit , J. C. Meyer , R. Erni , M. D. Rossell , C. Kisielowski , L. Yang , C.‐H. Park , M. F. Crommie , M. L. Cohen , S. G. Louie , A. Zettl , Science 2009, 323, 1705. 19325110 10.1126/science.1166999

[193] C. Tao , L. Jiao , O. V. Yazyev , Y.‐C. Chen , J. Feng , X. Zhang , R. B. Capaz , J. M. Tour , A. Zettl , S. G. Louie , H. Dai , M. F. Crommie , Nat. Phys. 2011, 7, 616.

[194] C. Casiraghi , A. Hartschuh , H. Qian , S. Piscanec , C. Georgi , A. Fasoli , K. S. Novoselov , D. M. Basko , A. C. Ferrari , Nano Lett. 2009, 9, 1433. 19290608 10.1021/nl8032697

[195] L. Zhou , L. Zhang , L Liao , M. Yang , Q. Xie , H. Peng , Z. Liu , Z. Liu , Acta Chim. Sinica 2014, 72, 289.

[196] J. Gao , J. Zhao , F. Ding , J. Am. Chem. Soc. 2012, 134, 6204. 22420470 10.1021/ja2104119

[197] H. Shu , X. Chen , X. Tao , F. Ding , ACS Nano 2012, 6, 3243. 22417179 10.1021/nn300726r

[198] D. V. Kosynkin , A. L. Higginbotham , A. Sinitskii , J. R. Lomeda , A. Dimiev , B. K. Price , J. M. Tour , Nature 2009, 458, 872. 19370030 10.1038/nature07872

[199] A. G. Cano‐Márquez , F. J. Rodríguez‐Macías , J. Campos‐Delgado , C. G. Espinosa‐González , F. Tristán‐López , D. Ramírez‐González , D. A. Cullen , D. J. Smith , M. Terrones , Y. I. Vega‐Cantú , Nano Lett. 2009, 9, 1527. 19260705 10.1021/nl803585s

[200] L. C. Campos , V. R. Manfrinato , J. D. Sanchez‐Yamagishi , J. Kong , P. Jarillo‐Herrero , Nano Lett. 2009, 9, 2600. 19527022 10.1021/nl900811r

[201] L. Jiao , L. Zhang , X. Wang , G. Diankov , H. Dai , Nature 2009, 458, 877. 19370031 10.1038/nature07919

[202] L. Tapaszto , G. Dobrik , P. Lambin , L. P. Biro , Nat. Nanotechnol. 2008, 3, 397. 18654562 10.1038/nnano.2008.149

[203] X. Wang , H. Dai , Nat. Chem. 2010, 2, 661. 20651729 10.1038/nchem.719

[204] Y. Huang , J. Wu , X. Xu , Y. Ho , G. Ni , Q. Zou , G. Koon , W. Zhao , A. H. Castro Neto , G. Eda , C. Shen , B. Özyilmaz , Nano Res. 2013, 6, 200.

[205] A. Basagni , F. Sedona , C. A. Pignedoli , M. Cattelan , L. Nicolas , M. Casarin , M. Sambi , J. Am. Chem. Soc. 2015, 137, 1802. 25582946 10.1021/ja510292b

[206] Z. Li , J. Yang , J. G. Hou , J. Am. Chem. Soc. 2008, 130, 4224. 18331034 10.1021/ja710407t

[207] D. W. Boukhvalov , M. I. Katsnelson , Nano Lett. 2008, 8, 4373. 19367969 10.1021/nl802098g

[208] C. Chen , M. Long , H. Wu , W. Cai , Sci. China Chem. 2013, 56, 354.

[209] L. Zhou , L. Zhang , L Liao , M. Yang , Q. Xie , H. Peng , Z. Liu , Z. Liu , Acta Chim. Sinica 2014, 72, 289.

[210] V. Georgakilas , M. Otyepka , A. B. Bourlinos , V. Chandra , N. Kim , K. C. Kemp , P. Hobza , R. Zboril , K. S. Kim , Chem. Rev. 2012, 112, 6156. 23009634 10.1021/cr3000412

[211] A. Muge , J. C. Yves , Jpn. J. Appl. Phys. 2011, 50, 070101.

[212] M. P. Zach , K. H. Ng , R. M. Penner , Science 2000, 290, 2120. 11118141 10.1126/science.290.5499.2120

[213] E. C. Walter , M. P. Zach , F. Favier , B. J. Murray , K. Inazu , J. C. Hemminger , R. M. Penner , ChemPhysChem 2003, 4, 131. 12619411 10.1002/cphc.200390022

[214] C. E. Banks , T. J. Davies , G. G. Wildgoose , R. G. Compton , Chem. Commun. 2005, 829. 10.1039/b413177k 15700054

[215] A. N. Patel , M. G. Collignon , M. A. O'Connell , W. O. Y. Hung , K. McKelvey , J. V. Macpherson , P. R. Unwin , J. Am. Chem. Soc. 2012, 134, 20117. 23145936 10.1021/ja308615h

[216] A. Anne , E. Cambril , A. Chovin , C. Demaille , C. Goyer , ACS Nano 2009, 3, 2927. 19769340 10.1021/nn9009054

[217] W. Yuan , Y. Zhou , Y. Li , C. Li , H. Peng , J. Zhang , Z. Liu , L. Dai , G. Shi , Sci. Rep. 2013, 3, 2248 23896697 10.1038/srep02248 PMC3727060

[218] W. Li , G. Zhang , M. Guo , Y.‐W. Zhang , Nano Res. 2014, 7, 518.

